# Evolutionary relationships among the snakelike pygopodid lizards: a review of phylogenetic studies of an enigmatic Australian adaptive radiation

**DOI:** 10.7717/peerj.11502

**Published:** 2021-06-29

**Authors:** W. Bryan Jennings

**Affiliations:** 1 Department of Evolution, Ecology, & Organismal Biology, University of California, Riverside, Riverside, California, United States of America; 2 Departamento de Vertebrados, Museu Nacional, Universidade Federal do Rio de Janeiro, Rio de Janeiro, RJ, Brasil

**Keywords:** Ancient hybridization, Lizards, Mito-nuclear discordance, Phylogenetics, Phylogenomics, Pygopodidae, Species complexes, Taxonomy, Testing monophyly, Tree rooting

## Abstract

Here, I review phylogenetic studies of the lizard family Pygopodidae, a group of 47 extant species that diversified in Australia and New Guinea. The goal of this study was to examine published phylogenetic and phylogenomic hypotheses on pygopodids to identify the strengths and weaknesses in our understanding of their phylogeny. Many parts of the pygopodid family tree are well established by multiple independent tree inferences including: (1) all multispecies genera (i.e., *Aprasia*, *Delma*, *Lialis*, *Pletholax*, and *Pygopus*) are monophyletic groups; (2) the root of the pygopodid tree is located along the branch leading to the *Delma* clade, thus showing that *Delma* is the sister group to all other pygopodid genera; (3) the *Aprasia repens* group, *Delma tincta* group, and several other groups of closely related species are demonstrated to be monophyletic entities; and (4) the monotypic *Paradelma orientalis* is the sister lineage to the *Pygopus* clade. Based on accumulated phylogenetic evidence, two taxonomic recommendations are given: *Paradelma* merits generic status rather than being subsumed into *Pygopus* as some earlier studies had suggested, and the monotypic *Aclys concinna* should be recognized as a member of *Delma* (following current practice) until future studies clarify its placement inside or outside the *Delma* clade. One chronic problem with phylogenetic studies of pygopodids, which has limited the explanatory power of many tree hypotheses, concerns the undersampling of known species. Although the continual addition of newly described species, especially over the past two decades, has been a major reason for these taxon sampling gaps, deficits in species sampling for ingroups and/or outgroups in several studies of pygopodid species complexes has confounded the testing of some ingroup monophyly hypotheses. Ancient hybridization between non-sister lineages may also be confounding attempts to recover the relationships among pygopodids using molecular data. Indeed, such a phenomenon can explain at least five cases of mito-nuclear discordance and conflicts among trees based on nuclear DNA datasets. Another problem has been the lack of consensus on the relationships among most pygopodid genera, an issue that may stem from rapid diversification of these lineages early in the group’s history. Despite current weaknesses in our understanding of pygopodid phylogeny, enough evidence exists to clarify many major and minor structural parts of their family tree. Accordingly, a composite tree for the Pygopodidae was able to be synthesized. This novel tree hypothesis contains all recognized pygopodid species and reveals that about half of the clades are corroborated by multiple independent tree hypotheses, while the remaining clades have less empirical support.

## Introduction

The richest lizard communities in the world are found in Australia where more than 40 species coexist together in single communities (Pianka, 1986; Morton & James, 1988). This spectacular diversity originated via adaptive and non-adaptive radiations involving lineages in five different lizard families (Jennings, Pianka & Donnellan, 2003; Jennings & Pianka, 2004; Rabosky et al., 2007; Collar et al., 2010; Collar, Schulte & Losos, 2011; Melville et al., 2011; Brennan, Bauer & Jackman, 2016; Brennan & Oliver, 2017; Brennan et al., 2020). Although evolutionary studies of Australian lizards lagged behind community ecological work of these species—the latter work having begun in the 1960s, molecular phylogeny-derived perspectives of this phenomenon have been catching up over the past two decades owing to advances in molecular phylogenetics and phylogenomics. Phylogenies inferred from DNA sequence data have yielded insights about the biogeographic and speciational histories (Jennings, Pianka & Donnellan, 2003; Jennings & Pianka, 2004; Rabosky et al., 2007; Melville et al., 2011; Brennan, Bauer & Jackman, 2016; Brennan & Oliver, 2017) and ecomorphological evolution (Collar et al., 2010; Collar, Schulte & Losos, 2011; Brennan et al., 2020; Gurgis et al., 2021) of these groups. Indeed, molecular phylogenies have played a central role in revealing the timing, tempo, and causes of these diversification events.

**Figure 1:**
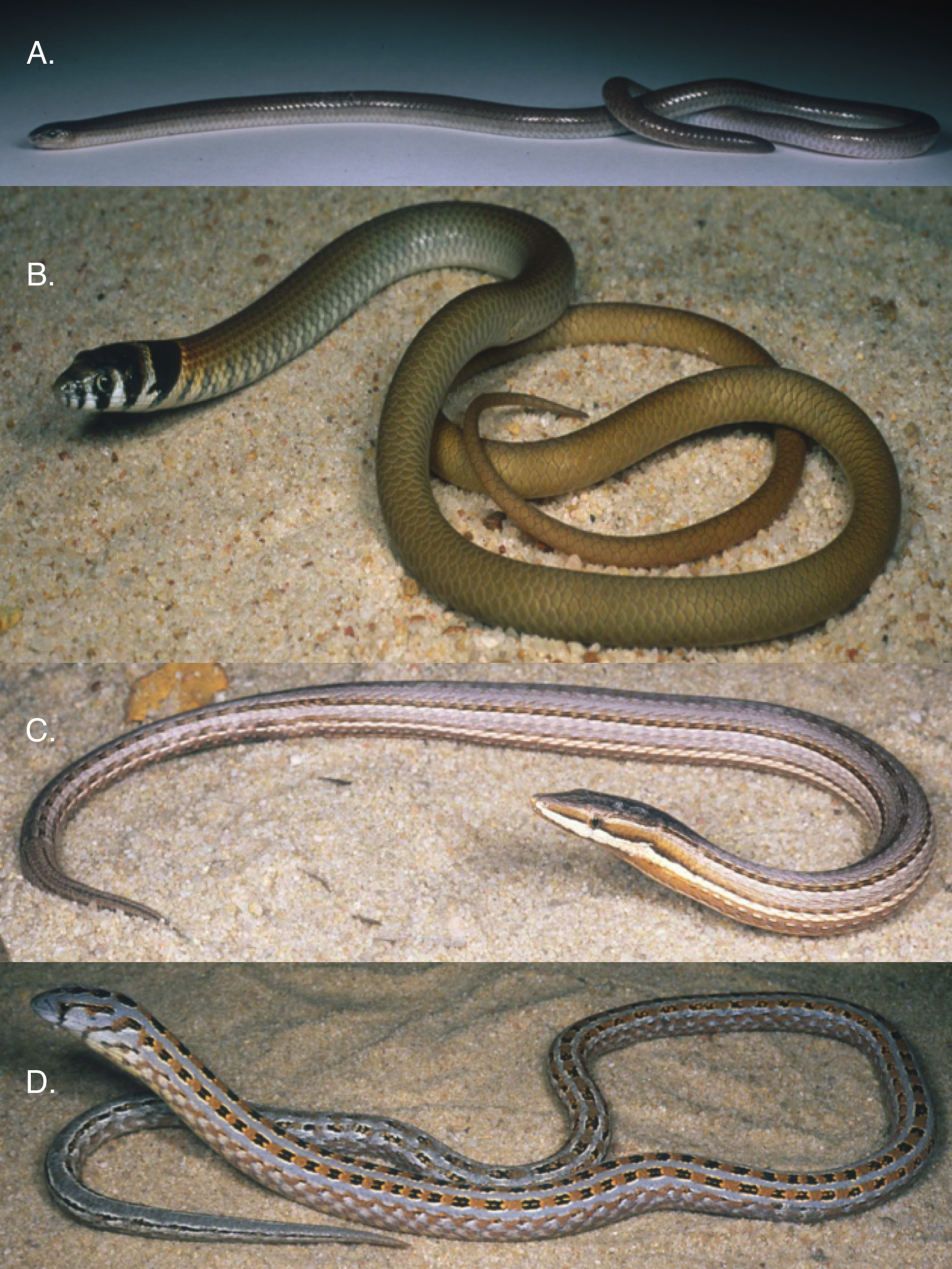
Examples of pygopodid lizards. (A*) Aprasia pseudopulchella*. (B) *Delma fraseri*. (C) *Lialis burtonis*. (D) *Pygopus lepidopodus*. All photos by W. Bryan Jennings.

A poorly known adaptive radiation is the lizard family Pygopodidae GA Boulenger, a group of elongate and limb-reduced lizards endemic to Australia and New Guinea (Kluge, 1974; Fig. 1). These lizards display an impressive range of body sizes and forms, which reflect their wide array of ecological specializations (Camp, 1923; Patchell & Shine, 1986; Greer, 1989; Webb & Shine, 1994; Garcia-Porta & Ord, 2013). Today, close to 50 extant pygopodid species are recognized (Brennan, Bauer & Jackman, 2016), and nearly a quarter of the group has only been discovered and described in the past two decades. Originally, eight pygopodid genera were described though the number of accepted genera remains controversial (e.g., Shea, 1987; Greer, 1989; Shea, 1993). These genera are: *Aclys* AG Kluge, *Aprasia* JE Gray, *Delma* JE Gray, *Lialis* JE Gray, *Ophidiocephalus* AHS Lucas and C Frost, *Paradelma* JR Kinghorn, *Pletholax* ED Cope, and *Pygopus* B Merrem. Although phylogenetic studies of pygopodids date back to the mid-1970s, the continual addition of new species over the years has confounded the efforts of researchers to infer a taxonomically complete phylogeny for the group.

Studies of pygopodid phylogenetics have focused on two different taxonomic scales. First, several studies were concerned with relationships among genera and majority of the species known at the time (Kluge, 1976; Jennings, Pianka & Donnellan, 2003; Brennan, Bauer & Jackman, 2016; Brennan & Oliver, 2017; Skipwith, Bi & Oliver, 2019). Second, other studies attempted to resolve relationships within putative species groups and to describe new species (James, Donnellan & Hutchinson, 2001; Maryan, Aplin & Adams, 2007; Oliver, Couper & Amey, 2010; Maryan, Bush & Adams, 2013; Maryan, How & Adams, 2013; Maryan, Adams & Aplin, 2015; Maryan et al., 2015; Kealley et al., 2020). “Species groups” are subjectively defined and presumably monophyletic groups (usually of closely-related congeneric species) that had been defined in previous taxonomic and phylogenetic studies on the basis of morphological and/or molecular evidence. In addition to varied taxonomic sampling across studies, different dataset types (i.e., morphology, allozymes, and DNA) and sizes (i.e., dozens of characters to thousands of DNA sequence loci) have been analyzed (Table 1). How can we use this collection of studies to synthesize a more complete picture of pygopodid phylogeny?

**Table 1:** Summary of phylogenetic work on pygopodid lizards between the years 1976–2020.

Study	Data	Aclys*	*Aprasia*	*Delma*	*Lialis*	Ophidiocephalus*	Paradelma*	Pletholax*	*Pygopus*	Genera	Species
Kluge (1976)	86 morphological characters	1	6	7	2	1	1	1	2	8	21
James, Donnellan & Hutchinson (2001)	35 allozyme loci	0	0	0	0	0	0	0	3 (1)	1 (1)	3 (1)
Jennings, Pianka & Donnellan (2003)	2 mtDNA, 1 nDNA loci	1	10	16	2	1	1	1	2	8	34
Maryan, Aplin & Adams (2007)	34 allozyme loci	0	0	7	0	0	0	0	0	1	7
Oliver, Couper & Amey (2010)	1 mtDNA locus	0	0	0	(1)	(1)	(1)	(1)	2(3)	1 (5)	2 (7)
Maryan, How & Adams (2013)	38 allozyme loci	0	5 (2)	0	0	0	0	0	0	1 (1)	5 (2)
Maryan, Bush & Adams (2013)	38 allozyme loci	0	6 (2)	0	0	0	0	0	0	1 (1)	6 (2)
Maryan, Adams & Aplin (2015)	38 allozyme loci	0	8 (6)	0	0	0	0	0	0	1 (1)	8 (6)
Maryan et al. (2015)	2 mtDNA, 4 nDNA loci	(1)	0	3(1)	0	0	0	0	0	1 (2)	3 (2)
Brennan, Bauer & Jackman (2016)	1 mtDNA, 4 nDNA loci	1	1	21	1	1	1	1	1	8	28
Brennan & Oliver (2017)	1 mtDNA, 6 nDNA loci	1	10	21	1	1	1	1	5	8	41
Skipwith, Bi & Oliver (2019)	4,248 UCE loci	0	6	14	2	0	1	1	4	6	28
Kealley et al. (2020)	1 mtDNA, 2 nDNA loci	0	(2)	(2)	(1)	(1)	(1)	2	(2)	1 (6)	2 (9)

**Note:**

Data and taxon sampling characteristics of each study are shown. Asterisks indicate monotypic genera in Kluge (1974). Numbers below each genus indicate the number of species in the designated ingroup while numbers in parentheses (if any) show the number of pygopodid species used to root the tree.

When competing tree hypotheses exist for a set of taxa, we can search for agreement among them. If any clades are observed in the majority of trees, then we can accept those clades as empirically corroborated clade hypotheses. These accepted clades can thus serve as building blocks in a new “composite tree” hypothesis for that organismal group (e.-g., Weiblen, Oyama & Donoghue, 2000). However, there is an important distinction between the types of trees involved in these comparisons. If multiple tree hypotheses are based partly or wholly on the same data, then such estimates of phylogeny are not independent of each other; that is, their topologies are correlated to each other to some degree thereby limiting the inferential strength of clade selections. In contrast, stronger evidence for a hypothesized clade’s existence comes from agreement among trees inferred from evolutionarily independent datasets. Although datasets comprised of morphological characters, allozyme loci, mitochondrial DNA (mtDNA) loci, and nuclear DNA (nDNA) loci are independent of each other, it is important to realize that mtDNA datasets—regardless of the number of mtDNA loci they contain—constitute a single independent dataset (Moore, 1995), whereas it is possible to have multiple independent nDNA datasets. This latter possibility arises because vertebrate genomes are comprised of thousands or more independent loci owing to the loci-unlinking effects of meiotic recombination and population demography (see Jennings, 2016, 2017 for reviews). Thus, before comparing a set of tree hypotheses for a group of taxa, it is imperative to know if the trees are independent of each other because that determination will impact the robustness of any clade selection conclusions.

It is also important to consider factors that could cause individual tree topologies to differ from the true species tree. For example, missing taxa in a phylogenetic analysis can lead to a tree that contains statistically significant but spurious clades (Heath, Hedtke & Hillis, 2008; Prum et al., 2015; Feng et al., 2020). Even gene trees inferred without topological errors can be incongruent with the group’s true species tree owing to the effects of ancestral polymorphisms, gene flow, and recombination (Nei, 1987; Moore, 1995; Maddison, 1997; Jennings & Edwards, 2005). However, today’s phylogenomic approaches—especially multispecies coalescent (MSC) methods for inferring species trees—can account for these factors thereby producing more accurate and precise species tree inferences (Edwards, 2009; Bryant et al., 2012; Faircloth et al., 2012; Lemmon, Emme & Lemmon, 2012). Thus, knowledge about the underpinnings of gene tree-species tree conflicts can be used to improve clade selection decisions in among-tree comparisons.

Evaluating statistical support for clades within single trees has been another standard component of phylogenetic analyses. The two main metrics for evaluating clade support in maximum parsimony (MP)/maximum likelihood (ML) and Bayesian inference (BI) trees have been non-parametric bootstrap proportions or “BP” (Felsenstein, 1985) and Bayesian posterior probabilities or “BPP,” respectively. When clades have BP values ≥ 70 (Hillis & Bull, 1993) or BPP values ≥ 0.95 (Huelsenbeck & Ronquist, 2001), they have strong statistical support. Although clades with low BP and BPP values likely low levels of phylogenetic signal in a dataset, it is important to remember that clades with high BP and BPP values can still be incongruent with the group’s true species tree (e.g., long-branch attraction; see Swofford et al., 1996). Parametric bootstrapping in a hypothesis-testing framework (Hillis, Mable & Moritz, 1996; Huelsenbeck, Hillis & Nielsen, 1996) is a second approach for ascertaining the support for a clade when only a single tree is considered. In this procedure, a tree score for the observed clade is compared to the score for a constraint tree containing the alternative clade hypothesis. A non-significant test result suggests that the data matrix contains low phylogenetic signal, whereas a significant result shows the observed clade is robustly supported by data. Another important within-tree clade analysis is the testing of monophyly hypotheses *(*see Swofford et al., 1996, pp. 477–478*)* of groups that are presumed to be monophyletic. Although this test is not statistically based as Swofford et al. (1996) noted, it is a critical preliminary step prior to inferring phylogenetic relationships within such groups. However, it is not uncommon to encounter a phylogenetic study—particularly involving species groups—that had employed weakened or more seriously flawed monophyly tests due to undersampling of taxa.

The efficacy of monophyly testing methods on single trees is dependent on ingroup sampling and how trees are rooted. The ideal methodology to conduct a test of monophyly consists of these steps: (1) sample all members of the putative monophyletic group (defined in a previous study); (2) sample all other species that could be confused with members of the presumed monophyletic groups (e.g., other congeners); and (3) root the tree using an outgroup that includes distant relatives (e.g., from another genus) or using molecular clock/midpoint methods (see Swofford et al., 1996; Huelsenbeck, Bollback & Levine, 2002; Felsenstein, 2004). The key is to ensure that the ingroup includes all members of the presumed species group plus other closely related species, and to select outgroup species that are unquestionably outside the ingroup. If the hypothetical species group is monophyletic in the tree, then evidence supporting that species group is obtained. However, even if appropriate outgroup species are used, this test of monophyly will be weakened if any species of the putative species group or closely related species are missing from the analysis because inclusion of any one of them could lead to rejection of the monophyly hypothesis.

Flawed monophyly tests arise in cases when an ingroup only consists of species belonging to the hypothetical species group and the outgroup is comprised of closely related species (i.e., typically congeners). In this study design, the ingroup is assumed to be monophyletic and therefore only the relationships among ingroup species can be inferred. This is because outgroups define their ingroups as monophyletic entities and so concluding that an ingroup is monophyletic because none of the outgroup samples were nested among the ingroup species is circular reasoning. Thus, only a partial test of monophyly can be performed (Swofford et al., 1996, p. 478). In this procedure, the entire tree (i.e., outgroup + ingroup species) is viewed as an unrooted tree. If more than one branch separates “outgroup” from “ingroup” species, then the monophyly hypothesis can be rejected. If, however, a single branch separates ingroup from outgroup samples, then the evidence is at best *consistent* with ingroup monophyly. Such a finding would only be consistent with a monophyletic ingroup because of the possibility that the root might be located within the ingroup (Swofford et al., 1996). However, if all ingroup and outgroup samples are treated as if they comprise one big ingroup, and the root position can be placed on this tree using a molecular clock (or midpoint method), or provided from an independent phylogenetic study, then evidence *supporting* ingroup monophyly can be obtained.

Here, I review the literature on pygopodid systematics that has accumulated over the past four decades. This review is directed at three audiences: herpetologists who conduct research on pygopodid and other gekkonid lizards, phylogeneticists and phylogeographers who study species complexes, and evolutionary biologists who are interested in the diversification of Australia’s modern fauna and flora. The main goals of this review are to: (1) evaluate the literature on the phylogenetic relationships among pygopodid lizards; (2) use this information to synthesize a composite phylogeny containing all recognized species; and (3) indicate the level of empirical support for each clade in the composite tree. The newly synthesized tree resulting from this study can thus serve as a framework for future phylogenomic and comparative studies of pygopodid lizards.

## Survey methodology

### General approach

A search for all peer-reviewed primary literature articles using the terms “Pygopodidae,” “phylogeny,” and “phylogenetics” was conducted in Google Scholar from all years until the present (November 2020). Studies that included the majority of valid pygopodid species or which were aimed at resolving species complexes were included in this review. Studies focused on higher-level relationships above the family Pygopodidae were excluded because they generally had sparse taxon sampling for pygopodids and did not include new data or taxa. The two exceptions to this rule were the studies by Brennan & Oliver (2017) and Skipwith, Bi & Oliver (2019) because they had inferred phylogenies for the majority of species in this family and were based in part or entirely on unpublished data.

This review is structured into four sections. First, a historical overview of the five major published studies of pygopodid phylogeny is provided. These studies were by Kluge (1976), Jennings, Pianka & Donnellan (2003), Brennan, Bauer & Jackman (2016), Brennan & Oliver (2017), and Skipwith, Bi & Oliver (2019). For brevity, they will hereafter be referred to as *K76*, *JP*&*D*, *BB*&*J*, *B*&*O*, and *SB*&*O*, respectively. Second, phylogenetic relationships within multispecies genera were evaluated using a set of explicit criteria (see below). Third, the set of published tree hypotheses for the Pygopodidae included in this review were evaluated to elucidate the most probable intergeneric relationships. Lastly, a composite phylogeny that includes all valid pygopodid species is then constructed based on the findings of this review.

### Choosing clades and accepted clade/lineage placements

I developed eight criteria for choosing clades, and placements of accepted clades and lineages in among-tree comparisons. These criteria are as follows:

**Criterion 1:** if clades of comparable species composition were observed in multiple trees, then the clade based on the largest number of characters (or loci) in the data matrix was chosen. This criterion is similar to one used by Weiblen, Oyama & Donoghue (2000).

**Criterion 2:** if clades of comparable species composition were found in multiple trees, then the clade containing the largest number of species was chosen. This criterion is similar to one used by Weiblen, Oyama & Donoghue (2000). Justification for this criterion comes from growing evidence that suggests increased taxon sampling in phylogenetic and phylogenomic analyses can increase the accuracy of inferred phylogenies (e.g., Heath, Hedtke & Hillis, 2008; Prum et al., 2015; Feng et al., 2020). This criterion is suitable for clades containing highly divergent lineages, whereas it is not appropriate for species groups because Poe (1998) concluded that incomplete taxon sampling may not impact phylogenetic accuracy for small (< 20 species) clades of closely-related species.

**Criterion 3:** if clades of comparable species composition were located in multiple trees, then the clade in the MSC tree based on a phylogenomic dataset was chosen over concatenation trees or trees based on few independent loci. Justification for this criterion comes from studies that showed coalescent based species trees tend to be more accurate than trees inferred from concatenated loci datasets (e.g., Jiang, Edwards & Liu, 2020).

**Criterion 4:** if a clade contained species largely not found in other trees, then that unique clade was accepted by default.

**Criterion 5:** if a clade was congruent with clades in all or the majority of trees, and those trees were based on the same dataset (i.e., different optimality criteria were used), then that clade was accepted.

**Criterion 6:** if a clade was found in all or the majority of trees, and those trees were based on evolutionarily independent datasets, then that clade was accepted.

**Criterion 7:** if placement of a clade or single-species lineage was the same in all or the majority of independent trees, then that placement was accepted. This criterion is similar to one used by Weiblen, Oyama & Donoghue (2000).

**Criterion 8:** if placements of an accepted/chosen clade or single-species lineage differed among independent trees, and none of the candidate placements was found in the majority of trees, then the placement suggested by the tree based on more characters (or loci) was accepted.

Several of these criteria are not mutually exclusive and thus multiple criteria could apply to particular cases. Independent corroboration was considered to be the strongest evidence supporting the existence of a particular clade and placement of an accepted monophyletic group or single species lineage in a tree (i.e., Criteria 6 and 7). Table 2 provides a summary of these eight criteria. Branch support statistics (i.e., BP and BPP values) were not used to evaluate the robustness of clade hypotheses because: (1) several published trees considered in this review did not include branch support values on their tree(s); and (2) as already mentioned, clades having statistically significant branch support values can still be incongruent with the true species tree topology for myriad reasons. The term “basal,” which is used throughout this paper, is here defined as the extant lineage that is sister to a clade of species under consideration.

**Table 2:** Summary of criteria used for choosing monophyletic group hypotheses and accepted clade/lineage placements in comparisons of multiple trees.

Criterion	Description
1	Clades based on datasets with more characters (or more loci) are preferred over clades based on smaller-sized datasets
2	Clades with more species are preferred over clades containing fewer species (does not apply to species groups)
3	MSC trees are preferred over the concatenation trees (or trees based on single loci)
4	Clades with unique species compositions are accepted (no alternative clade hypotheses exist)
5	Clades found in the majority of trees based on the same data are preferred
6	Clades found in the majority of trees based on independent data are preferred
7	If placements of accepted clades and single-species lineages are the same in all or the majority of independent trees, then those placements are preferred
8	If placements of accepted clades or single-species lineages differ among independent trees, then the placements suggested by the trees based on more characters (or loci) are preferred

**Note:**

Criteria 6 and 7 result in the strongest inferences because they are based on corroboration by multiple independent trees. Note, Weiblen, Oyama & Donoghue (2000) listed criteria that are similar to Criteria 1, 2, and 7 in this table. See main text for detailed descriptions of each criterion.

### Synthesizing a composite tree for the Pygopodidae

The findings in this review were used to synthesize a composite tree hypothesis for all recognized pygopodid species. Thus all selected clades and single-species lineages were grafted onto a single tree in their most likely placements based on the evidence. Because these clades received variable amounts of empirical support, it was possible to assign a level-of-support designation to each clade to reflect the evidence for each clade’s existence. The levels of clade support were: (1) “low support” = clade was supported by a single independent tree; (2) “medium support” = clade was found in an MSC tree based on hundreds or more genome-wide loci, or was corroborated by two independent trees but missing taxa or conflicting evidence raises uncertainty about that clade’s existence; and (3) “high support” = clade was supported by multiple independent trees, or the clade was comprised of two species that were formerly described as a single species.

## Historical overview of pygopodid phylogenetics

### Pygopodid phylogeny from Kluge (1976)

**Figure 2:**
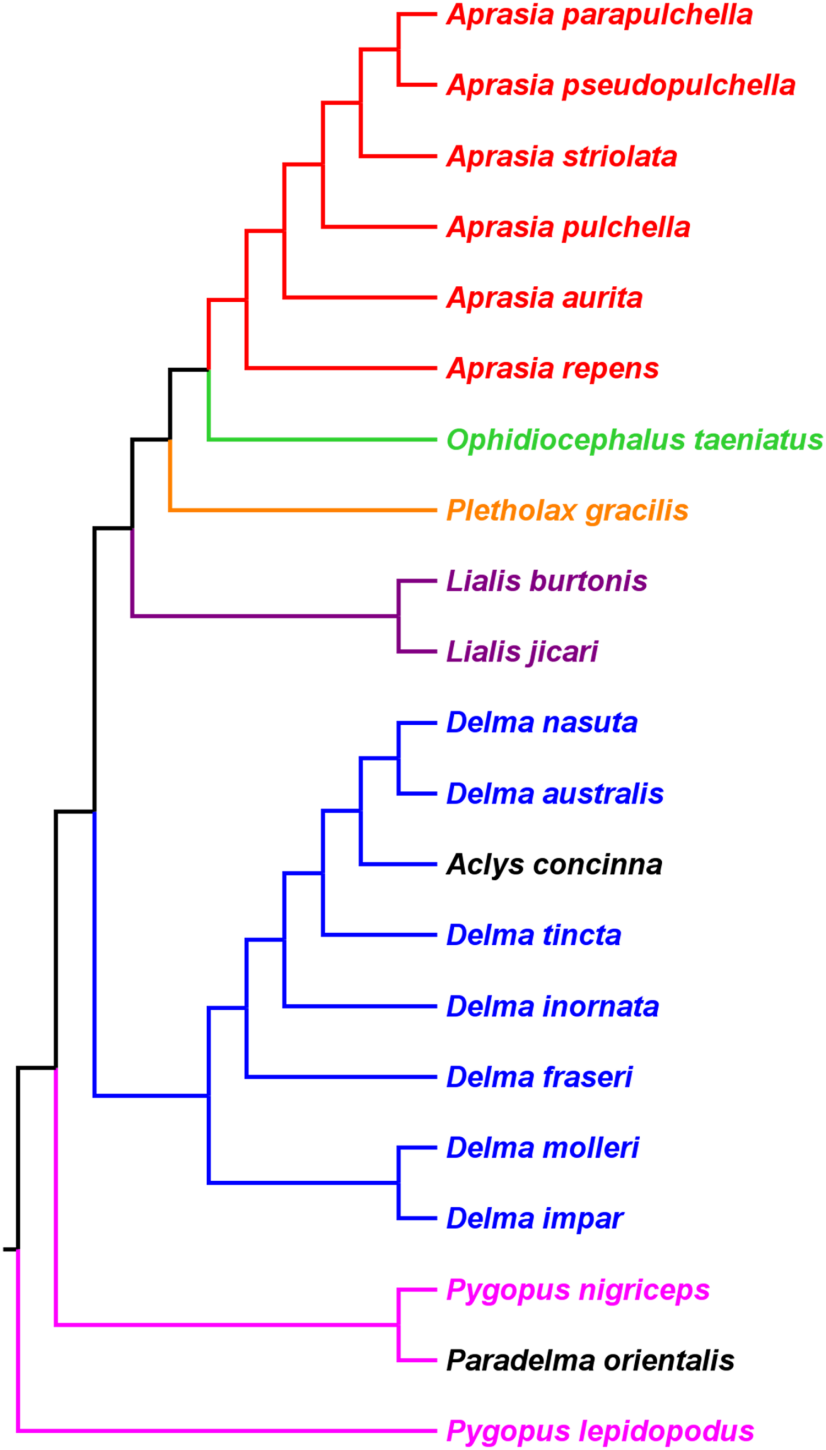
Phylogenetic hypothesis of the Pygopodidae inferred from 86 morphological characters. Shown is a cladogram rooted along the branch leading to *Pygopus lepidopodus* (see main text). Notice that the placements of *Aclys concinna* and *Paradelma orientalis* (in black) cause *Delma* and *Pygopus*, respectively, to not be monophyletic genera. The root position also causes Pygopus to be paraphyletic. After Fig. 9 in Kluge (1976).

Kluge (1976) used morphological data to infer the relationships among 21 of the 30 known pygopodid species at that time, a sample that included representatives of all eight genera. The phylogenetic results of that study showed *Aprasia* and *Lialis* to each be monophyletic, but neither *Delma* nor *Pygopus* were monophyletic (Fig. 2). Surprisingly, the monotypic *Aclys concinna* AG Kluge was placed inside *Delma* while the monotypic *Paradelma orientalis* A Günther was the sister species to *Pygopus nigriceps* JG Fischer, an arrangement that caused *Pygopus* to become paraphyletic (Fig. 2). Note that the location of the tree’s root also caused *Pygopus* to be paraphyletic. Kluge (1976, pp. 26–27*)* rooted the tree with *P. lepidopodus* Lacépède after concluding that this taxon was the sister lineage to all other extant pygopodids.

To reconcile pygopodid generic nomenclature with these phylogenetic results, *K76* subsumed *Aclys* and *Paradelma* into the genera *Delma* and *Pygopus*, respectively. Although these actions resulted in a revised taxonomic scheme consisting of six genera (i.e., *Aprasia*, *Delma*, *Lialis*, *Ophidiocephalus*, *Pletholax*, and *Pygopus*), *K76* (p. 68*)* noted that *Aclys concinna* and *Paradelma orientalis* each had unique scalation characters that made them distinct from all other extant pygopodids. Accordingly, Kluge recommended that both species still be recognized as monotypic taxa, albeit as sub-genera, a conclusion that was reflected in the revised classification for the family (*K76*, p. 69*)*. Thus to keep in mind Kluge’s observation that *Aclys* and *Paradelma* are morphologically unique amongst pygopodids, I provisionally refer to these two species in the text below and associated tree figures as *Delma* (*Aclys*) *concinna* and *Pygopus* (*Paradelma*) *orientalis*. At the conclusion of this review, I make taxonomic recommendations for both species in light of all phylogenetic evidence to date.

### Pygopodid phylogeny from Jennings, Pianka & Donnellan (2003)

#### MtDNA and nDNA phylogenetic hypotheses, and problems with finding the root

**Figure 3:**
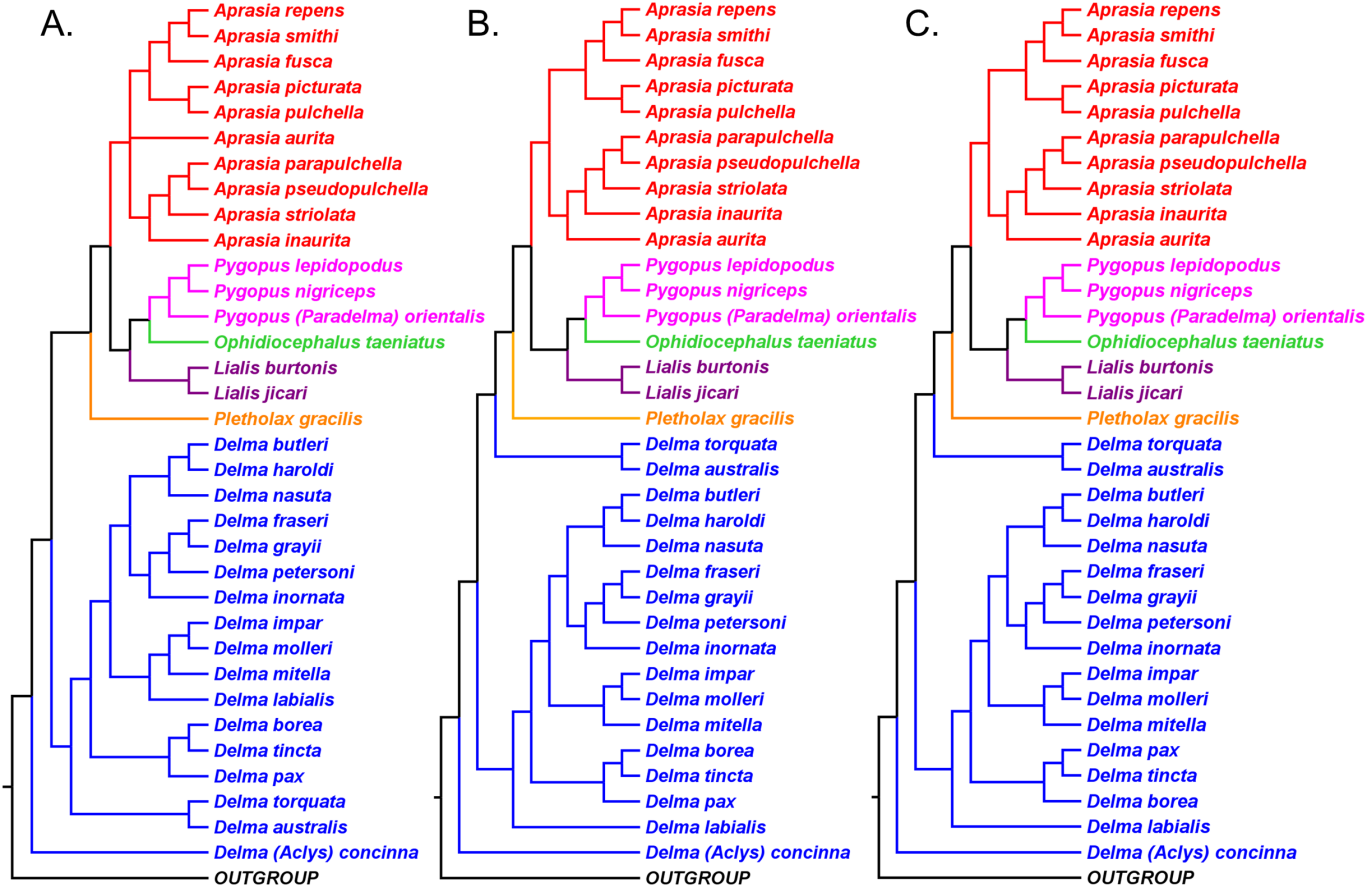
Phylogenetic hypotheses of the Pygopodidae inferred from two concatenated mtDNA (*16S* and *ND2*) genes. (A) Maximum parsimony cladogram. (B) Maximum likelihood cladogram. (C) Bayesian inference cladogram. All trees were rooted using two diplodactyline gecko species in the outgoup. Classification scheme for genera follows Kluge (1976) but subgeneric names of *Aclys* and *Paradelma* are also shown in parentheses. Note that *Aprasia fusca* is now recognized as *A. rostrata* (Maryan, Bush & Adams, 2013). After Fig. 2 in Jennings, Pianka & Donnellan (2003).

**Figure 4:**
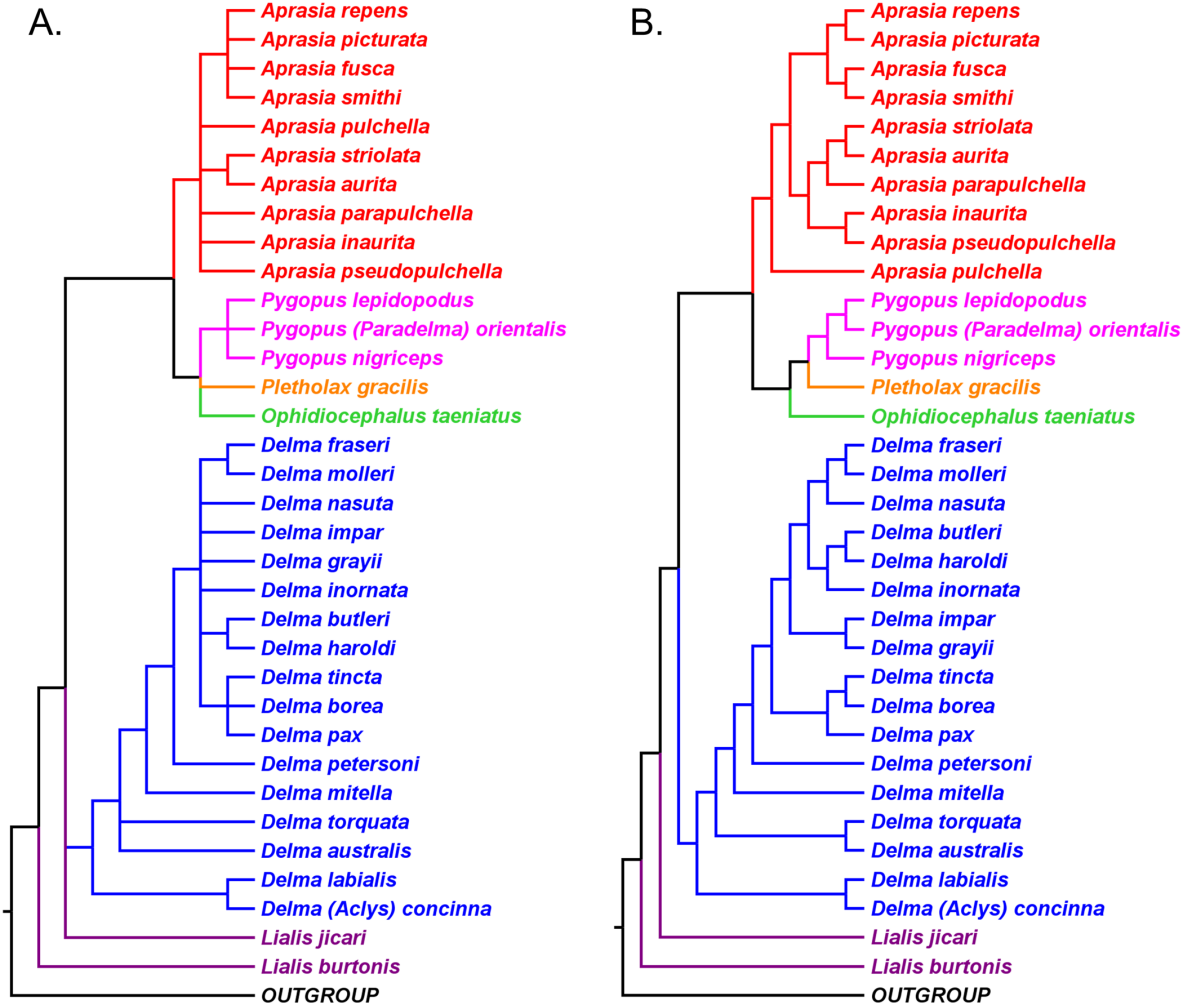
Phylogenetic hypotheses of the Pygopodidae inferred from the nuclear *C-mos* gene. (A) Maximum parsimony/maximum likelihood cladogram. (B) Bayesian inference cladogram. All trees were rooted using two diplodactyline gecko species in the outgoup. Note that *Aprasia fusca* is now recognized as *A. rostrata* (Maryan, Bush & Adams, 2013). After Fig. 4 in Jennings, Pianka & Donnellan (2003).

**Figure 5:**
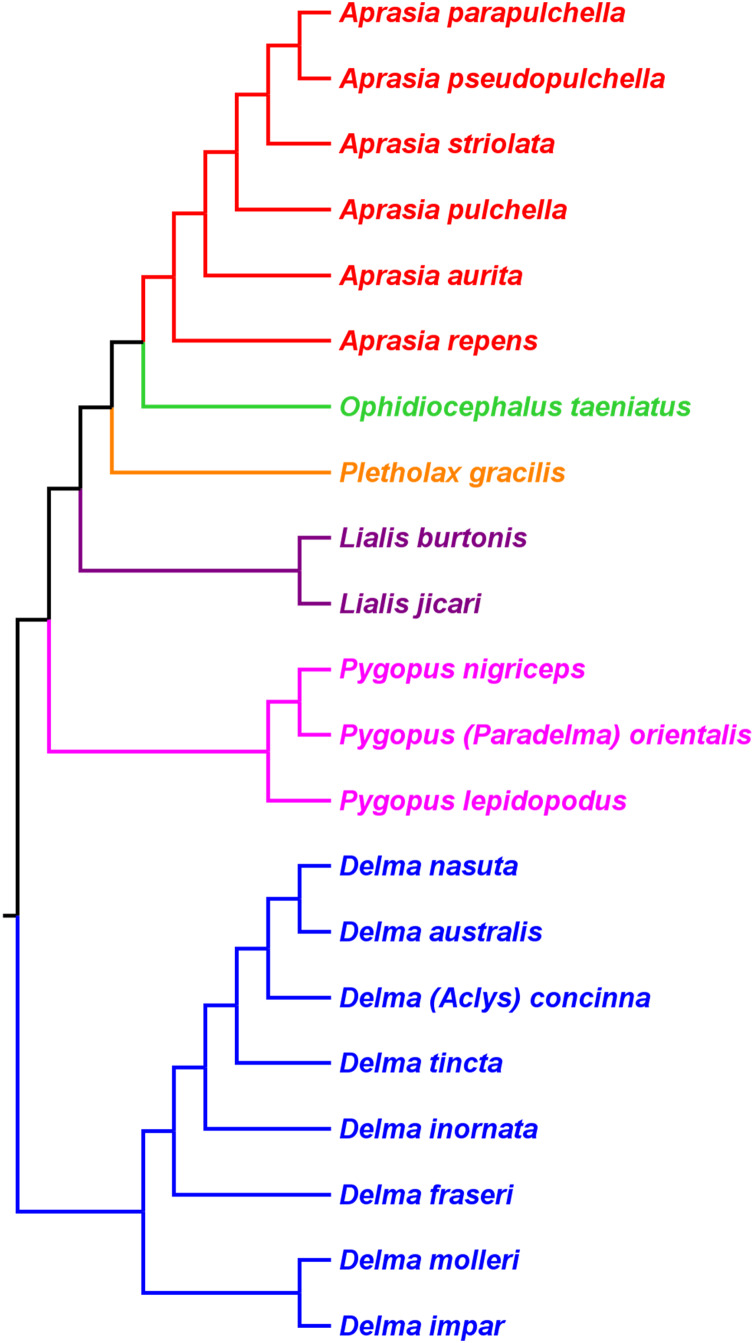
Re-rooted tree of the Pygopodidae based on 86 morphological characters. This tree has the same unrooted topology as the tree in Fig. 2 but was rooted along the branch leading to the *Delma* clade (see main text). Modified version of Fig. 9 in Kluge (1976).

**Figure 6:**
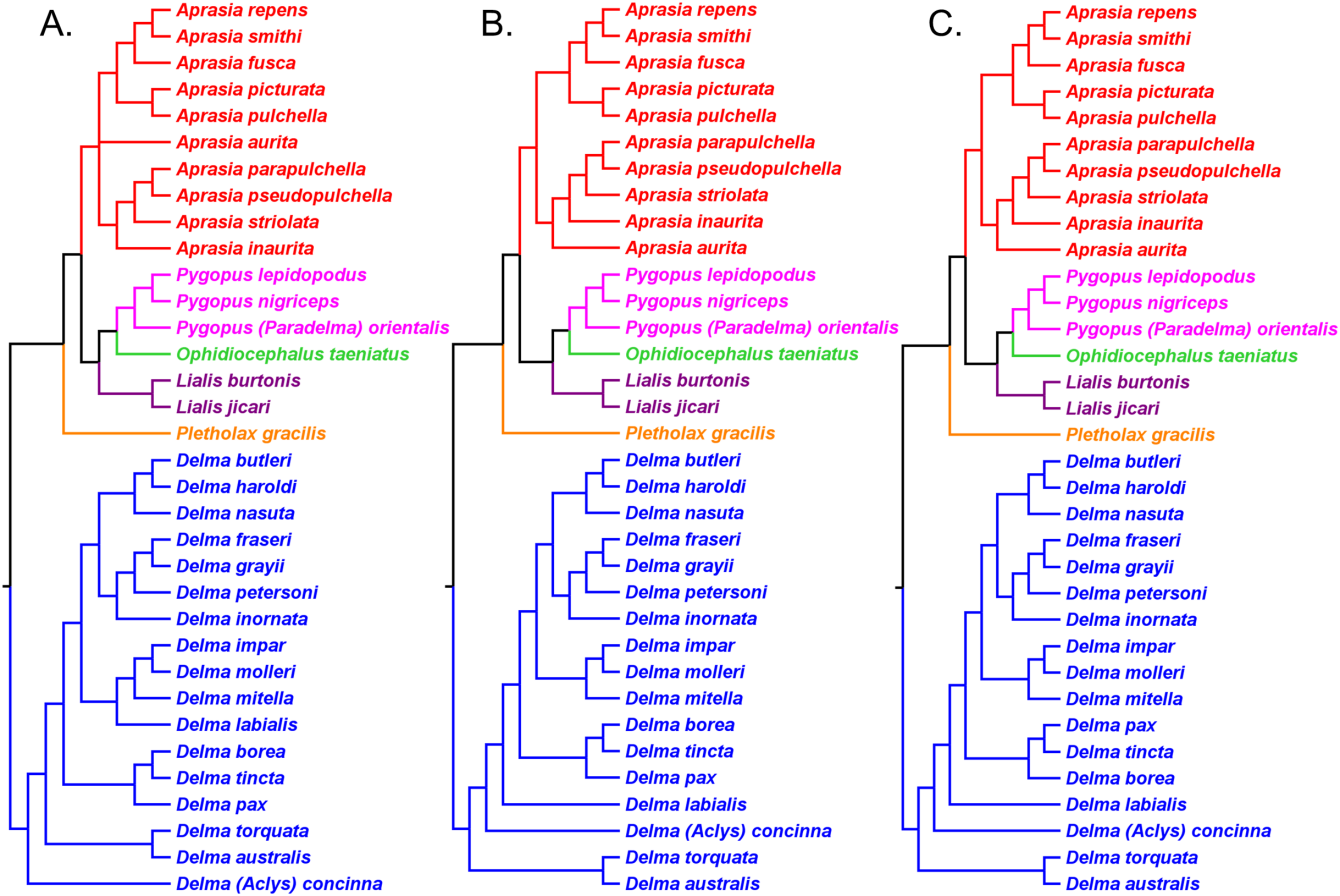
Re-rooted mtDNA trees of the Pygopodidae inferred from concatenated *16S* and *ND2* genes. (A) Maximum parsimony cladogram. (B) Maximum likelihood cladogram. (C) Bayesian inference cladogram. All trees have the same unrooted topologies found in Fig. 3 but were rooted along the branch leading to the *Delma* clade (see main text). Modified version of Fig. 2 in Jennings, Pianka & Donnellan (2003).

Jennings, Pianka & Donnellan (2003) inferred the phylogenetic relationships among 34 of the 38 pygopodid species described by that time, including all genera, using two mtDNA (*16S* and *ND2*) genes (1,706 base pairs (bp)) and one nDNA (*C-mos*) gene (373 bp). Maximum Parsimony, ML, and BI analyses of the mtDNA dataset led to the recovery of monophyletic *Aprasia*, *Lialis*, and *Pygopus* in each resulting tree (Fig. 3). However, regardless which optimality criterion was used to infer trees, the outgroup lineage consistently attached itself to the *Delma* (*Aclys*) *concinna* branch, causing *concinna* to be the sister lineage to all other extant pygopodids and thus rendering *Delma* paraphyletic (Fig. 3). In analyses of the nDNA dataset, *Aprasia*, *Delma*, and *Pygopus* were all monophyletic, but *Lialis* was paraphyletic owing to the outgroup attaching itself to the *Lialis burtonis* JE Gray branch (Fig. 4). Although the root location selected by the outgroup lineage remained consistent among analyses of the same dataset type, its position was not stable between trees based on different datasets (Figs. 3 and 4). Further root instability was observed when two additional root locations were observed in trees inferred from concatenated mtDNA + nDNA data and concatenated molecular + morphological data (see Figs. 5 and 6 in *JP*&*D*)

#### Resolution of the rooting problem in the pygopodid tree

Owing to the observations that the outgroup lineage was rooting the pygopodid tree in various tree locations, it was suspected that the DNA sequences of the ingroup may have been too divergent from the outgroup sequences to enable accurate rooting of the trees. Indeed, a highly divergent outgroup may root the tree in a random and often wrong location in the ingroup (Swofford et al., 1996). Although the outgroup consisted of appropriate taxa—two diplodactyline gecko species, which are close relatives to pygopodids (Kluge, 1987; Donnellan, Hutchinson & Saint, 1999; *B&O*; *SB&O*), this outgroup was evidently too divergent from the ingroup to be of use for finding the tree’s root location. In view of this, *JP*&*D* concluded that the lack of consensus for a single hypothesized root location was due to long-branch attraction between a highly divergent outgroup combined with long ingroup branches.

To identify the correct root location, *JP*&*D* excluded outgroup species from their datasets and then used a molecular clock to root each of their trees. The molecular clock approach consistently identified the branch leading to the *Delma* clade as the root location in mtDNA, nDNA, and combined mtDNA + nDNA analyses. Because each tree contained 65 possible rooting locations (i.e., branches), there was only a 1 in 65 chance of two independent trees finding agreement on the root’s location. Accordingly, those results strongly suggested that *Delma* must be the sister group to all other extant pygopodid genera.

#### Re-rooted pygopodid trees show monophyletic multispecies genera

**Figure 7:**
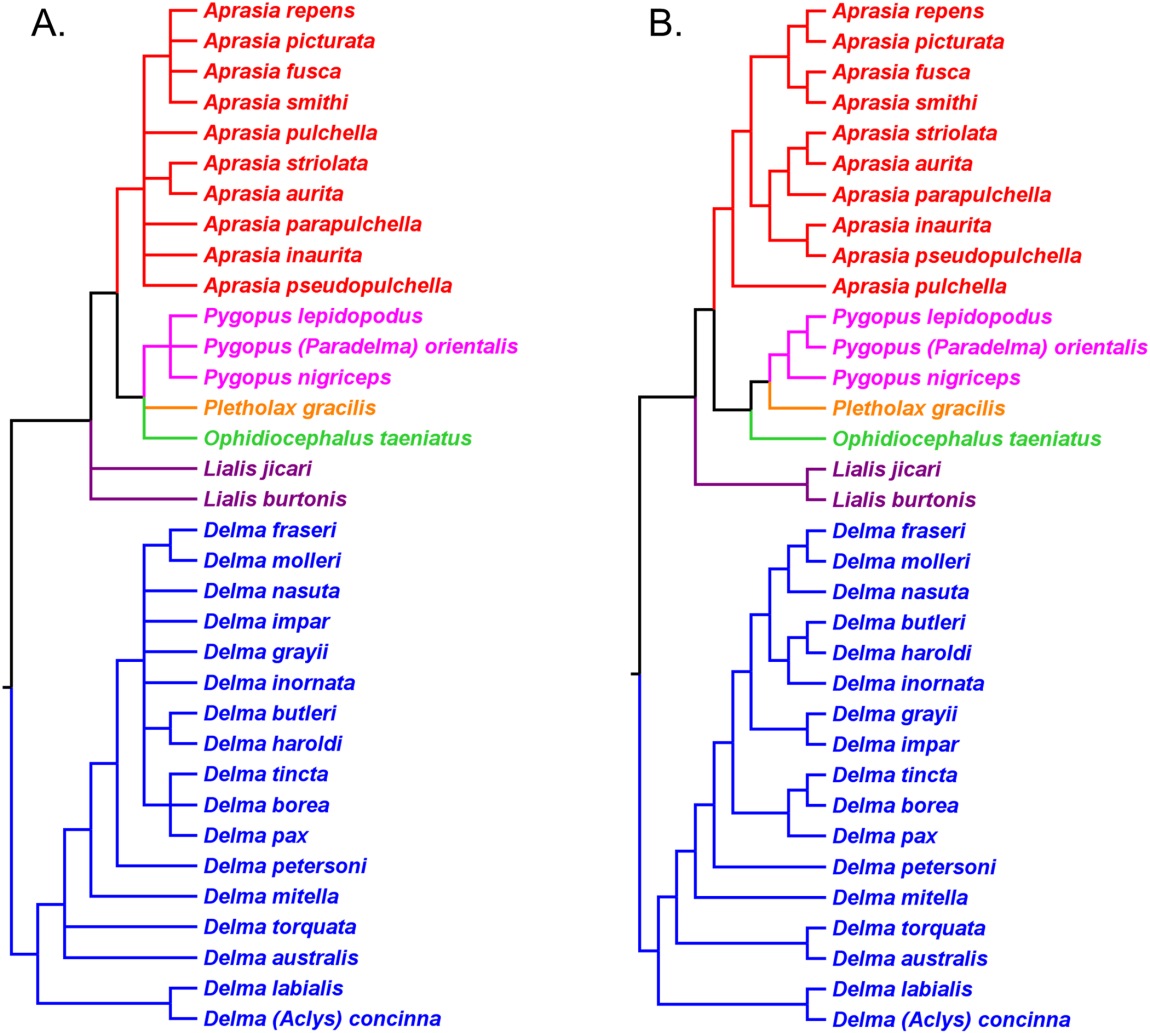
Re-rooted nDNA trees of the Pygopodidae inferred from the *C-mos* gene. (A) Maximum parsimony/maximum likelihood cladogram. (B) Bayesian inference cladogram. All trees have the same unrooted topologies found in Fig. 4 but were rooted along the branch leading to the *Delma* clade (see main text). Modified version of Fig. 4 in Jennings, Pianka & Donnellan (2003).

If we reposition the root on *K76*’s morphology tree to be on the branch leading to the *Delma* clade, then all multispecies genera—*Aprasia*, *Delma*, *Pygopus*, and *Lialis*—are monophyletic as would be expected (Fig. 5). Similarly, re-rooting *JP*&*D’s* mtDNA and nDNA trees also produced monophyletic genera with only two exceptions (Figs. 6 and 7). The exceptions were the MP and ML nDNA trees, each of which showed non-monophyletic *Lialis* (Fig. 7A). However, these unexpected results are likely due to insufficient phylogenetic signal in the *C-mos* gene sequences because the BI nDNA tree did show a monophyletic *Lialis* (Fig. 7B). Still, it is remarkable that the nDNA dataset in this case, which was comprised of only 373 nucleotide sites, was able to recover monophyletic groups for all multispecies pygopodid genera. The combined results of the re-rooted morphology and molecular trees support monophyly of these genera in two ways. First, if we look at each tree individually, we can see that each multispecies genus is monophyletic. Second, monophyly of these genera is corroborated across trees based on three independent datasets (i.e., morphology, mtDNA, and nDNA; Criterion 6; Table 2).

### Pygopodid phylogeny from Brennan, Bauer & Jackman (2016)

#### Monophyletic *Delma*, mtDNA introgression, and support for the root

**Figure 8:**
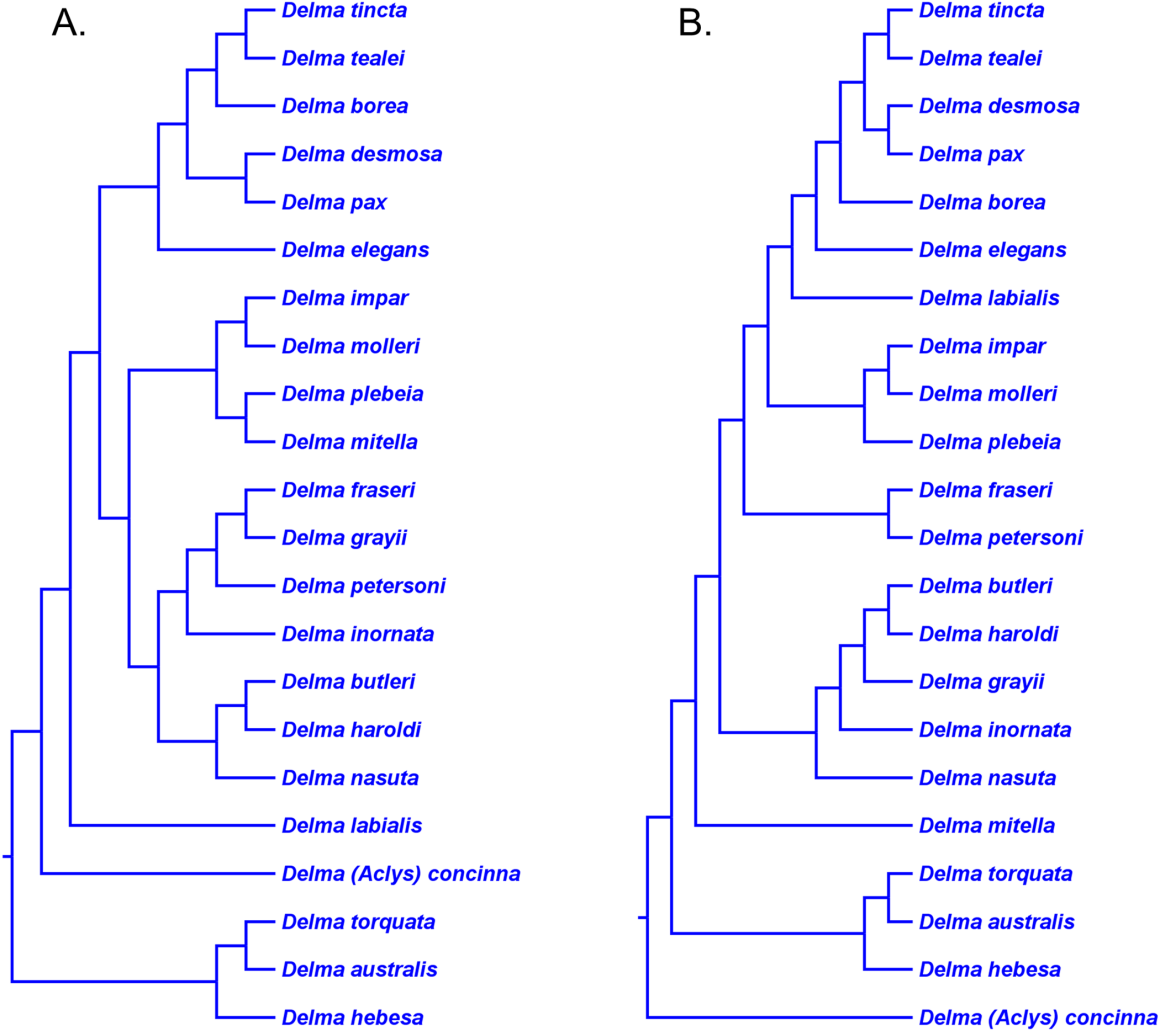
Phylogenetic hypotheses of the the genus *Delma* based on mtDNA and nDNA data. (A) Maximum likelihood/Bayesian inference cladogram inferred from an mtDNA (*ND2* gene and flanking tRNAs) sequences. (B) Maximum likelihood/Bayesian inference cladogram inferred from four concatenated nDNA (*C-mos, DYNLL1, RAG1*, and *MXRA5*) genes. Trees were rooted using a variety of outgroup taxa (not shown). Modified version of Fig. 1 in Brennan, Bauer & Jackman (2016).

Brennan, Bauer & Jackman (2016) conducted a molecular phylogenetic study of pygopodids based on a 1,480 bp mtDNA fragment (*ND2* and associated tRNAs) and four nDNA genes (i.e., *C-mos, DYNLL1*, *RAG1*, and *MXRA5*) that had a concatenated length of 3,019 bp. Because the nDNA trees in this study were based in part on the same *C-mos* sequences that were used to infer an nDNA tree in *JP*&*D*, these trees are not completely independent of each other. In addition to inferring phylogenies from two evolutionarily independent datasets, two other strengths of their study included: (1) sampling all 22 known species of *Delma* including *D*. (*Aclys*) *concinna*, and (2) sampling one member from each of the other pygopodid genera including *Pygopus (Paradelma*) *orientalis*. Although not shown in their trees, the authors evidently included several non-pygopodid lizard species in their outgroup (see Table 1 in *BB*&*J*), which permitted them to infer the root location of the pygopodid tree and test the monophyly of *Delma*. In their Fig. 1, which is reproduced here in Fig. 8, *BB*&*J* showed a pair of trees inferred from their mtDNA and nDNA datasets. For their mtDNA dataset, these authors used ML and BI to infer trees, while they used ML and BI to infer trees from their concatenated nDNA dataset and a Bayesian species tree program to infer a species tree based on three of their four nuclear genes, treating them as independent loci. Monophyly of *Delma* was demonstrated within and between their mtDNA and nDNA trees (Criterion 6; Table 2)—a significant finding considering that all recognized species in *Delma* had been sampled.

The topology of the *Delma* clade based on their mtDNA data was largely in agreement with the mtDNA trees in *JP&D (*compare Figs. 6 and 8A). However, these mtDNA trees conflicted with the nDNA trees in *BB&J (*compare Figs. 6, 8A and 8B). Brennan, Bauer & Jackman (2016) attributed much of the discordance between mtDNA and nDNA trees to three presumed cases of ancient mitochondrial introgression. According to these workers, the hypothesized hybridization events took place between *D. fraseri* J.E. Gray and *D. grayii* A. Smith, between *D. plebeia* C.W. De Vis and *D. mitella* G.M. Shea, and between *D. borea* A.G. Kluge and the most recent common ancestor to *D. tincta* C.W. De Vis and *D. tealei* B. Maryan, K.P. Aplin & M. Adams (Fig. 8). Another significant finding in *BB*&*J* was that their mtDNA and nDNA trees independently showed *Delma* to be the sister clade to all other pygopodid genera thereby corroborating the root position hypothesis by *JP*&*D*.

#### Hypothesized intergeneric relationships in pygopodids and four groups in *Delma*

Brennan, Bauer & Jackman (2016) preferred their nDNA trees (Fig. 8B) instead of their mtDNA trees (Fig. 8A) ostensibly for two main reasons. First, several relationships involving species of *Delma* in the mtDNA haplotype trees appeared to be artifacts of hybridization as just discussed. Second, the nDNA tree was based on a larger dataset consisting of several nuclear genes. These authors also inferred a Bayesian molecular clock tree (“time tree”) from their concatenated nDNA data. Of importance to our discussion here are not the divergence times suggested by that Bayesian time tree, but rather three other features of that tree.

**Figure 9:**
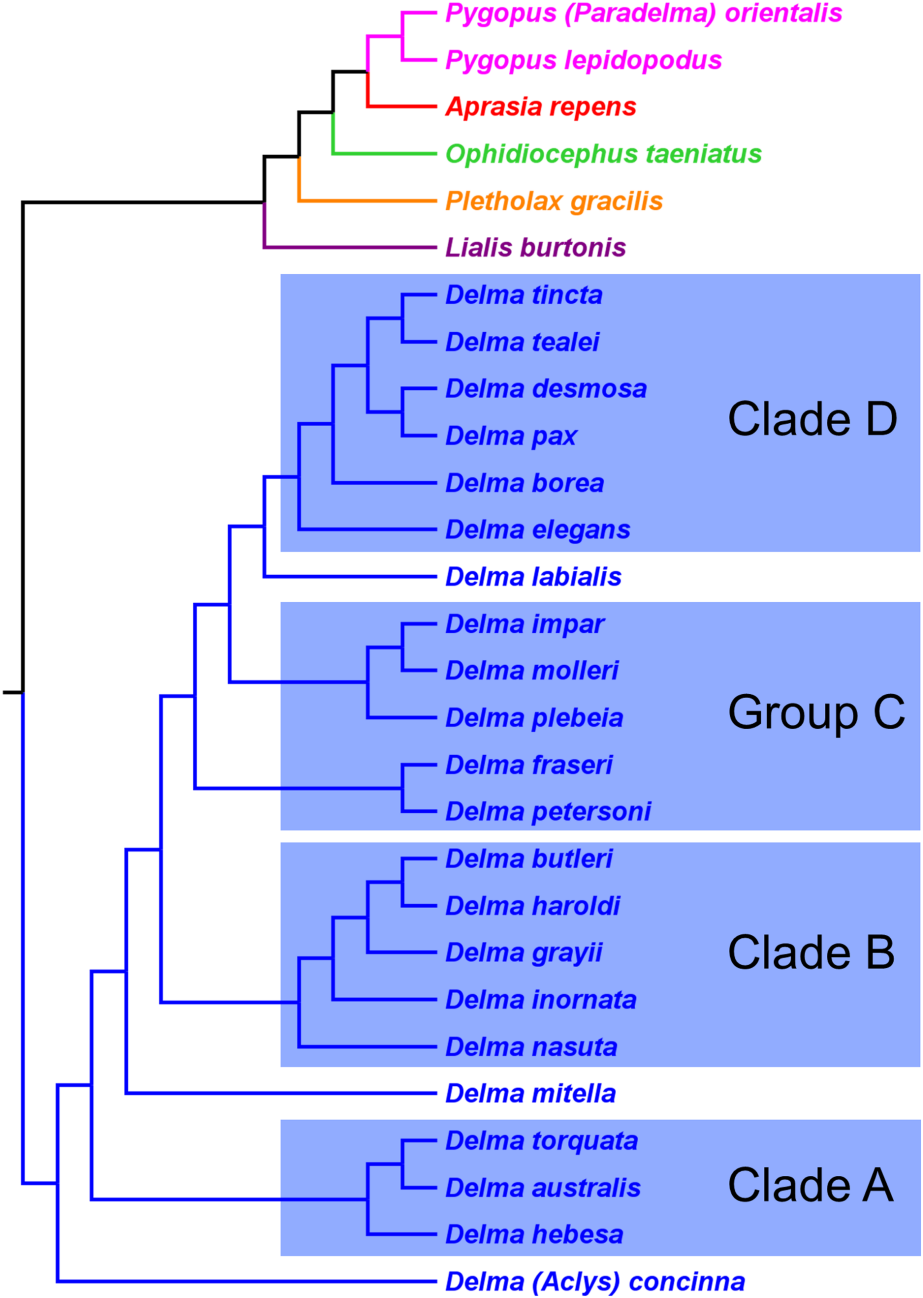
Phylogenetic hypothesis of the Pygopodidae inferred from four concatenated nDNA (*C-mos, DYNLL1, RAG1*, and *MXRA5*) genes. Shown is a cladogram based on the topology of a Bayesian time tree. Tree was rooted via a molecular clock. Groups shaded in blue boxes represent Clades A, B, D, and Group C (see main text). Modified version of Fig. 2 in Brennan, Bauer & Jackman (2016).

First, the topology of their time tree presented a hypothesis for the relationships among all eight original pygopodid genera (Fig. 9). Unfortunately, however, their intergeneric relationship hypothesis differed from hypotheses suggested by the re-rooted morphology tree (Fig. 5) of *K76*, as well as the re-rooted mtDNA (Fig. 6) and nDNA (Fig. 7) trees of *JP*&*D*. We will return to this topic of pygopodid intergeneric relationships below. A second important feature of their nDNA time tree is that it highlighted four groups of species within *Delma* that *BB*&*J* had defined based on phylogenetic, morphological, and biogeographical considerations: Clade A, Clade B, Group C (a paraphyletic group), and Clade D (Fig. 9). These groups will provide us with frameworks for considering the phylogenetic relationships within the speciose genus *Delma* (see below). Note that three other species—*D. (Aclys) concinna*, *D. mitella*, and *D. labialis* GM Shea—could not be assigned to any of these groups in the nDNA trees in *BB*&*J*, possibly because each one represents a highly divergent single-species lineage. We will further discuss this topic below.

### Pygopodid phylogeny from Brennan & Oliver (2017)

#### Inference of a pygopodid tree containing 41 species

Brennan & Oliver (2017) used a Bayesian approach to infer a fossil-calibrated phylogeny for the Pygopodoidea, a superfamily that comprises the families Carphodactylidae, Diplodactylidae, and Pygopodidae. Their tree hypothesis, which included 41 of the 46 extant species of pygopodids known at that time, was based on a concatenated DNA dataset consisting of one mtDNA (*ND2*) and six nDNA (i.e., *C-mos*, *DYNLL1*, *PDC*, *RAG1*, *RAG2*, and *ACM4*) genes. This dataset was largely compiled with DNA sequences published in prior studies. Accordingly, their tree is not independent of the mtDNA trees in *JP*&*D*, *BB*&*J*, and Oliver, Couper & Amey (2010), nor is it independent of the nDNA trees in *JP*&*D* and *BB*&*J*.

**Figure 10:**
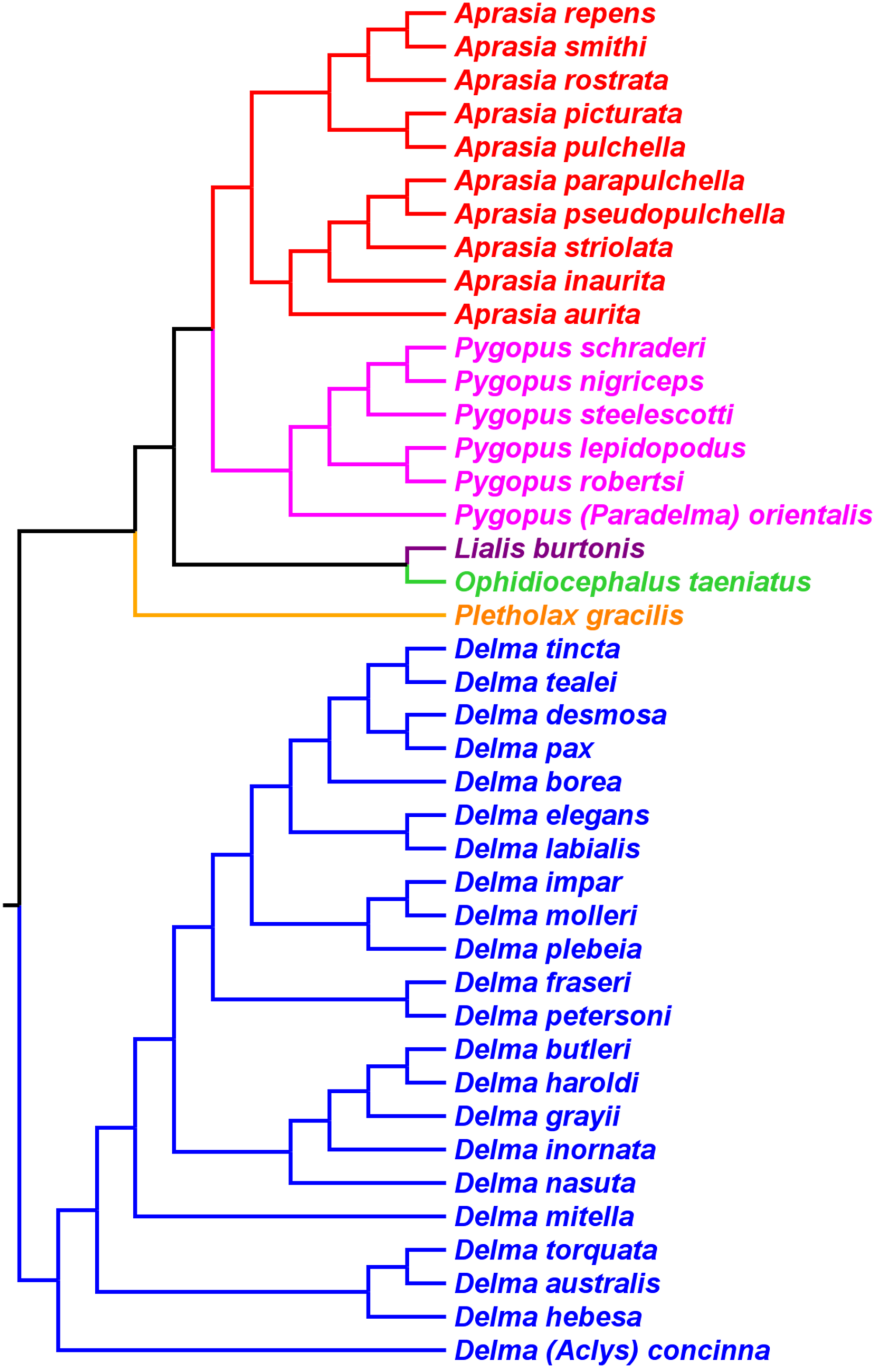
Phylogenetic hypothesis of the Pygopodidae inferred from one mtDNA (*ND2*) gene and six nDNA (*C-mos, DYNLL1, PDC, RAG1*, *RAG2*, and *ACM4*) genes. Shown is a cladogram based on the topology of a Bayesian time tree. Root placement was determined by an outgroup comprised of geckos in the Carphodactylidae and Diplodactylidae (not shown). Modified version of Fig. 1 in Brennan & Oliver (2017).

As we can see in Fig. 10, the tree in *B&O* displays a number of features that match up well with trees in earlier studies: (1) *Aprasia*, *Delma*, and *Pygopus* were recovered as monophyletic groups (see Figs. 5, 6, 7 and 9); (2) root was placed along the *Delma* branch; (3) inferred relationships among ten species of *Aprasia* were identical to those found in mtDNA trees of *JP*&*D* (compare Figs. 6 and 10); (4) inferred relationships among all twenty-two species of *Delma* were nearly identical to those found in the nDNA tree of *BB*&*J* (compare Figs. 8B and 10); and (5) the relationships among the five species of *Pygopus* were nearly identical to those found in the mtDNA tree of Oliver, Couper & Amey (2010; see below). As intimated, however, these similarities are not by coincidence because they clearly stem from the use of common DNA sequences. Indeed, examination of taxon sampling by gene in *B&O* shows substantial variation in taxon sampling among genes. For instance, the relationships within their *Aprasia* clade, and to a large extent within their *Pygopus* clade, must be solely due to the mtDNA portion of their data matrix because there were insufficient nDNA sequences in their data to account for their results. Similarly, the *Delma* clade inference in *B&O* must be largely due to the nDNA portion of their data matrix (i.e., *DYNLL1* and *RAG1* genes) due to the lack of sequences for the other nDNA genes as well as the mtDNA gene. Because most interspecific relationships in the tree inferred by *B&O* can be seen in earlier published trees considered here, I will limit discussion of this tree to cases whereby relationships in it differed from other trees (see below).

### Pygopodid phylogeny from Skipwith, Bi & Oliver (2019)

#### UCE trees corroborated monophyly of multispecies genera and the tree root

**Figure 11:**
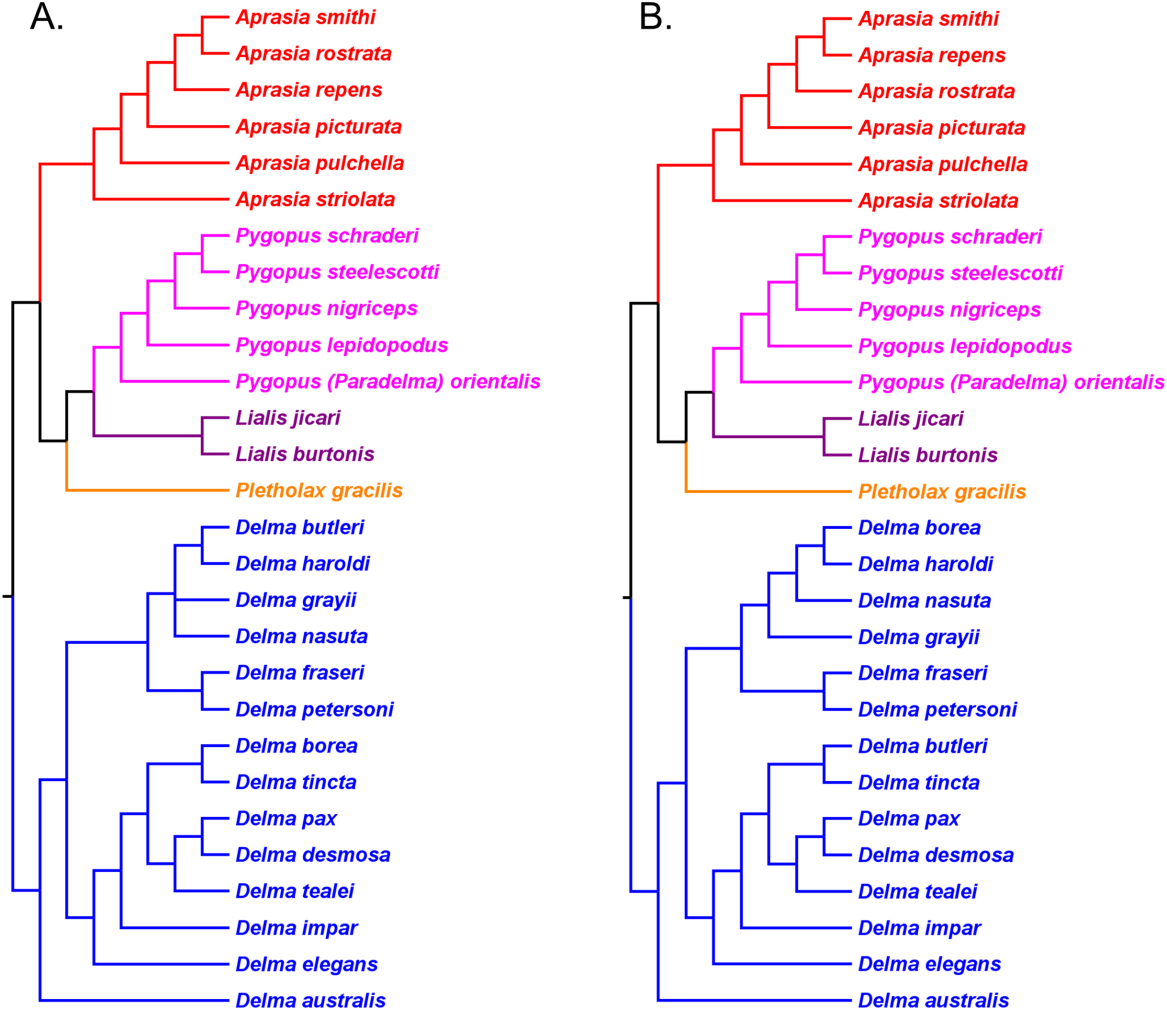
Phylogenetic hypotheses of the Pygopodidae based on 4,268 ultraconserved elements (UCE) loci. (A) Multispecies coalescent (MSC) species tree cladogram. (B) Maximum likelihood concatenated UCE loci cladogram. Root placement was determined by a large number of gecko species (not shown). Modified version of Fig. 4 in Skipwith, Bi & Oliver (2019).

Skipwith, Bi & Oliver (2019) inferred phylogenetic relationships among 28 species of pygopodids using 4,268 ultraconserved element (UCE) loci. Their taxon sampling of the group included six species of *Aprasia*, 14 species of *Delma*, both species of *Lialis*, the monotypic *Pletholax gracilis* Schlegel, and five species of *Pygopus* including *P*. (*Paradelma*) *orientalis*. Skipwith, Bi & Oliver (2019) used two different approaches to infer a phylogeny for this group: an MSC approach and an ML analysis of their concatenated UCE loci dataset. Their MSC species tree (hereafter MSC-UCE tree) and concatenated UCE tree showed *Aprasia*, *Delma*, *Lialis*, and *Pygopus* to be monophyletic groups as expected (Fig. 11). Moreover, both trees provided additional corroboration for the location of the root along the branch leading to *Delma* (Fig. 11). Although the intergeneric relationships were identical between their two trees, the absence of *D. (Aclys) concinna* and *Ophidiocephalus* precluded the inference of intergeneric comparisons involving the original eight pygopodid genera recognized in Kluge (1974).

The aforementioned studies showed widespread agreement on several major structural parts of the pygopodid family tree. First, each multispecies genus was generally found to be monophyletic within each tree. Second, when comparisons are made of trees based on independent datasets, we further see that monophyly of all multispecies genera is corroborated (Criterion 6; Table 2). And third, multiple independent trees support the placement of the root along the branch leading to *Delma* (Criterion 6; Table 2). We will now review the relationships within each multispecies genus.

## Intrageneric relationships in the pygopodidae

### Genus *Aprasia*

In a taxonomic monograph of the Pygopodidae, Kluge (1974) recognized nine species of *Aprasia*. The known species richness of this genus has since grown to 14 species after an additional five species were discovered in Western Australia (Storr, 1978; Smith & Henry, 1999; Maryan, Bush & Adams, 2013; Maryan, How & Adams, 2013; Maryan, Adams & Aplin, 2015). There have been several attempts to place these species in a phylogenetic tree though none of the studies have placed all of them into a single tree. Phylogenetic studies by *K76* and *SB&O* each included six species from this genus, but only three of those species were common to both studies (Figs. 5 and 11). Jennings, Pianka & Donnellan (2003) sampled the nine species analyzed by *K76* and *SB&O* plus one additional species (Fig. 6). Maryan, Bush & Adams (2013), Maryan, How & Adams (2013) and Maryan, Adams & Aplin (2015) also conducted phylogenetic analyses involving species of *Aprasia*, but their investigations were focused on resolving relationships within the *A. repens* species complex.

**Figure 12:**
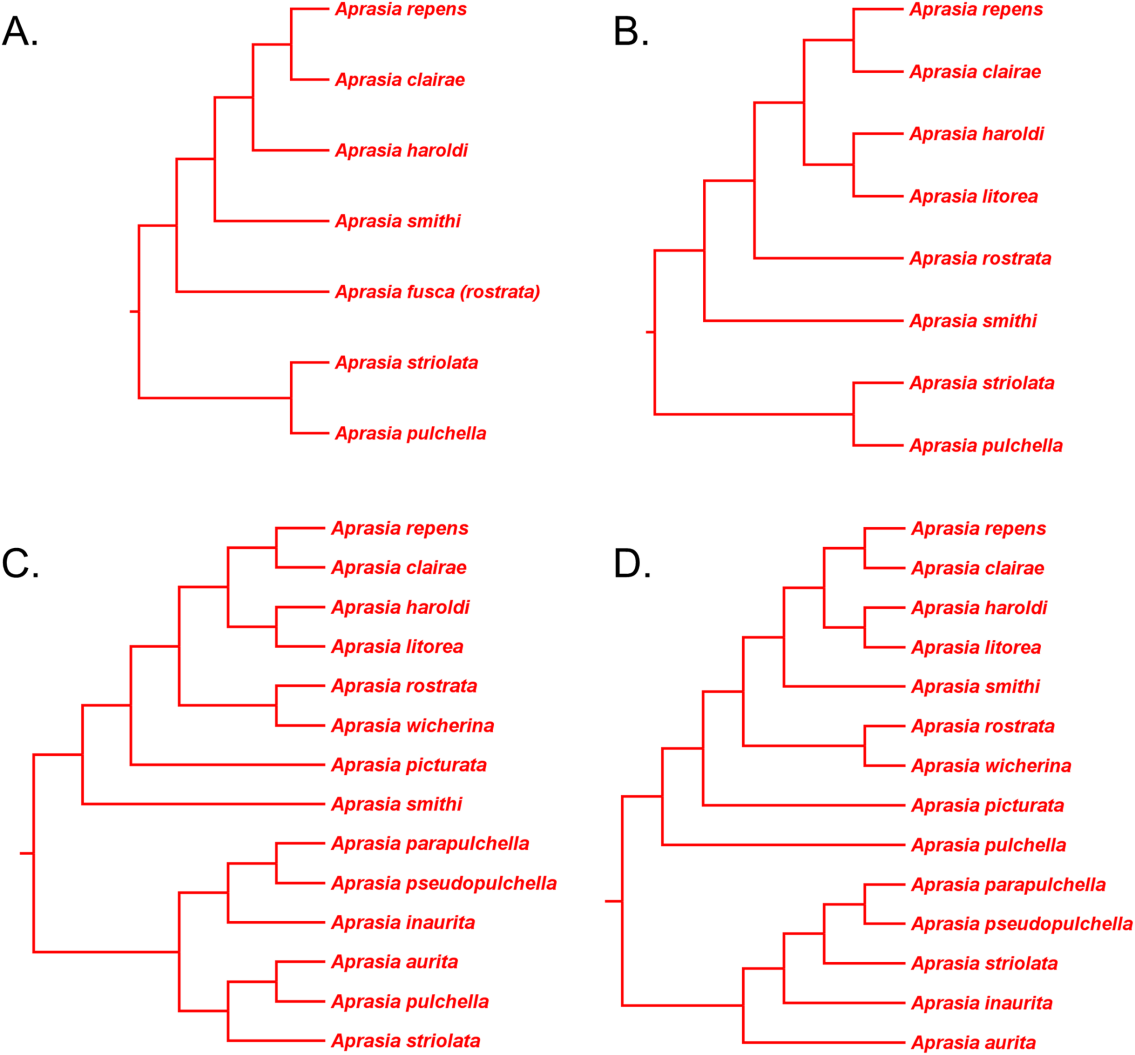
Phylogenetic hypotheses of the *Aprasia repens* species group and a novel phylogenetic hypothesis for *Aprasia*. (A) Neighbor-joining tree based on 38 allozyme loci (after Fig. 3 in Maryan, How & Adams, 2013). Note that *Aprasia fusca* is now recognized as *A. rostrata* (Maryan, Bush & Adams, 2013). (B) Neighbor-joining tree based on 38 allozyme loci (after Fig. 3 in Maryan, Bush & Adams, 2013). (C) Neighbor-joining tree based on 38 allozyme loci (after Fig. 3 in Maryan, Adams & Aplin, 2015). Each tree was rooted with between two and six congeneric species believed to be outside the *A. repens* group (i.e., *A. striolata*, *A. pulchella*, *A. parapulchella*, *A. pseudopulchella*, *A. inaurita*, and *A. aurita*). (D) Novel phylogenetic hypothesis for *Aprasia* based on morphology, allozymes, mtDNA, and nDNA (this study).

The mtDNA trees in *JP*&*D* showed *Aprasia* split into two geographical clades: a five-species “southeastern clade” largely found in southeastern Australia, and a five-species “western clade” found exclusively in Western Australia (see Fig. 12 in *JP*&*D*). The southeastern group contained *A. parapulchella* A.G. Kluge, *A. pseudopulchella* A.G. Kluge, *A. striolata* C. Lütken, *A. inaurita* A.G. Kluge, and *A. aurita* A.G. Kluge, while the western group was comprised of *A. pulchella* J.E. Gray, *A. picturata* L.A. Smith & J. Henry, *A. repens* D.B. Fry, *A. smithi* G.M. Storr, and *A. fusca* G.M. Storr, L.A. Smith & R.E. Johnstone. Note that *A. fusca* is now *A. rostrata* H.W. Parker following the taxonomic revision by Maryan, Bush & Adams (2013). Two species belonging to the southeastern clade, *A. striolata* and *A. inaurita*, also have populations located along the southern coastline of Western Australia (Kluge, 1974; Storr, Smith & Johnstone, 1990; Ehmann, 1992). Species in the western clade are distributed along the west coast of Australia, on nearby continental islands, and in scattered adjacent inland localities (Storr, Smith & Johnstone, 1990; Smith & Henry, 1999).

### Southeastern Australian *Aprasia* group

Of the five species comprising the southeastern Australian mtDNA clade, a sub-clade in this group comprised of ((*A. parapulchella*, *A. pseudopulchella*), *A. striolata*) was also observed in the morphology tree in *K76* (Fig. 5). Accordingly, this three-species group represents an independently corroborated clade (Criterion 6; Table 2). Another southeastern Australian species, *A. inaurita*, was placed as the sister lineage to the *A. parapulchella*/*A. pseudopulchella*/*A. striolata* clade in *JP*&*D*’s mtDNA tree, while the fifth southeastern species, *A. aurita*, was placed as the sister lineage to all other members of the southeastern clade (Fig. 6). Because no other trees contained *A. inaurita*, *A. aurita*, *A. parapulchella*, *A. pseudopulchella*, and *A. striolata* together, I regard the placements of *A. inaurita* and *A. aurita* in the mtDNA tree as the current hypothesis for their relationships (Criterion 4; Table 2). Although the placement of *A. pulchella* in the morphology tree suggests that this species may instead be closer to the mtDNA-defined southeastern *Aprasia* group (Fig. 5), independent phylogenetic evidence shows *A pulchella* to be a member of the Western Australian group (see below).

### Western Australian *Aprasia* group

The western Australian mtDNA haplotype group contained the three-species sub-clade ((*A. repens*, *A. smithi*), *A. fusca* [now *A. rostrata*]) and a sister lineage consisting of an *A. pulchella*/*A. picturata* sister species pair (Fig. 6). Both UCE trees in *SB&O* placed *A. striolata* as the sister lineage to a clade comprised of only endemic Western Australian *Aprasia* species (Fig. 11). In contrast to the mtDNA tree, the MSC-UCE tree in *SB&O* positioned *A. pulchella* as the sister lineage to the other exclusively western species of *Aprasia* while *A. picturata* was, in turn, the sister lineage to the ((*A. rostrata*, *A. smithi*), *A. repens*) clade (Fig. 11A). Their concatenated UCE tree was concordant with their MSC-UCE tree except the positions of *A. repens* and *A. rostrata* were reversed; that is, *A. repens* was the sister species to *A. smithi* (Fig. 11B). We will first evaluate the monophyly of the *A. repens* species group before we address the conflict between mtDNA and UCE trees in terms of the placements of *A. picturata* and *A. pulchella* in the Western Australian clade.

### Testing monophyly of the *Aprasia repens* species group

Storr, Smith & Johnstone (1990) defined the *Aprasia repens* species group based on morphological characters and species’ distributional patterns. This species complex initially included five Western Australian species (*A. repens*, *A. smithi*, *A. haroldi* GM Storr, *A. fusca*, and *A. rostrata*), which are distributed along the western to northwestern coasts of Western Australia, on nearby islands, and in adjacent inland localities. Membership of this group was later expanded to include additional species after the discoveries of *A. picturata*, *A. clairae* B. Maryan, R.A. How & M. Adams, *A. litorea* B. Maryan, B.G. Bush & M. Adams, and *A. wicherina* B. Maryan, M. Adams & K.P. Aplin. However, because Maryan, Bush & Adams (2013) had subsumed *A. fusca* into *A. rostrata*, the group now contains eight, not nine, species (Maryan, Adams & Aplin, 2015). Nonetheless, this species group contains eight of the fourteen recognized species in *Aprasia*. While all members of this group are only found in Western Australia, three other *Aprasia* species—*A. pulchella*, *A. striolata*, and *A. inaurita*, which are presumably outside this group, also occur in Western Australia. In Western Australia, these three species are only found in the extreme southwestern and southern parts of this region. Although members of the *A. repens* group are united together on morphological and geographical similarities, the question begs: does any molecular phylogenetic evidence exist that supports the monophyly of this eight-species group?

We begin our evaluation of this question using mtDNA trees in *JP*&*D* because they contained the majority of species in this genus including four species from the *A. repens* group (i.e., *A. picturata*, *A. fusca (A. rostrata)*, *A. smithi*, *A. repens*; Fig. 6). Notice that although *A. fusca (A. rostrata)*, *A. smithi*, and *A. repens* formed a monophyletic group, the presence of the *A. picturata*/*A. pulchella* sister mtDNA haplotype group is evidence against the *A. repens* group monophyly hypothesis (Fig. 6). However, evidence that supports monophyly of these same four species of the *A. repens* group comes from the UCE trees in *SB&O* (Fig. 11). Although this constitutes evidence supporting the monophyly hypothesis, this conclusion is tempered by the fact that the UCE trees included only half the species found in the *A. repens* group.

Several allozyme phylogenetic studies of the *A. repens* group provide additional opportunities to evaluate this monophyly hypothesis. In two allozyme studies, Maryan, How & Adams (2013) and Maryan, Bush & Adams (2013) defined their ingroups to include most of the species belonging to the *A. repens* group while their outgroups included two other species of *Aprasia* (Figs. 12A and 12B). Given their taxon-sampling schemes, we can partially test the monophyly of the *A. repens* group with their trees. Treating these two trees as if they were unrooted, we can see that a single branch separates the ingroups from outgroups in both cases (Figs. 12A and 12B). Accordingly, we cannot reject monophyly of the *A. repens* group and we can conclude that both trees constitute evidence consistent with *A. repens* group monophyly. Although these trees do not by themselves support monophyly of the *A. repens* group, recall that if extrinsic evidence about the tree’s root is available, then a stronger inference about monophyly can be made. And such evidence exists—both mtDNA and UCE trees show the root being located outside the clade containing *A. repens* group members (Figs. 6 and 11). Taken together, the evidence from the two unrooted allozyme trees with two independent estimates of the root’s location, support monophyly of the *A. repens* group.

In a later allozyme study, Maryan, Adams & Aplin (2015) inferred a complete phylogeny for the *A. repens* group (Fig. 12C). There are two notable aspects of their tree, which differentiates it from the trees of Maryan, How & Adams (2013) and Maryan, Bush & Adams (2013). First, these workers sampled all eight members of the presumed *A. repens* group. Second, they included all other known species of *Aprasia* in their outgroup. However, because they rooted their tree using only congeneric species, we are limited to performing a partial test of monophyly for the *A. repens* group. Looking at their tree in unrooted form, we see again that only a single branch separates the *A. repens* group cluster from the outgroup cluster and thus we cannot reject the monophyly hypothesis (Fig. 12C). By itself, this tree provides evidence that is consistent with a monophyletic *A. repens* group. But if we combine this result with other information that suggests the root position of the *Aprasia* clade is outside the *A. repens* group (see Figs. 6 and 11), then the combined findings provide compelling single-tree evidence supporting the group’s monophyly.

Recall that the mtDNA trees in *JP*&*D* had placed an *A. pulchella*/*A. picturata* clade as the sister group to a clade comprised of *A. fusca* (*A. rostrata*), *A. smithi*, and *A. repens* (Fig. 6), whereas both UCE trees in *SB&O* showed *A. pulchella* as the sister lineage to a monophyletic *A. repens* group (Fig. 11). Several lines of evidence favor the topology found in the UCE trees over the mtDNA tree topology with respect to the placements of *A. pulchella* and *A. picturata*. First, an allozyme tree (Fig. 12C) also suggests that *A. picturata* is a closer relative to the clade containing *A. rostrata*, *A. smithi*, and *A. repens* than it is to *A. pulchella*—thereby corroborating the UCE results (Criterion 7; Table 2). Second, the placements of *A. pulchella* and *A. picturata* suggested by the UCE trees are favored because they are based on far larger datasets compared to the mtDNA tree (Criterion 8; Table 2). Third, because one of the UCE trees was an MSC tree, this tree hypothesis is preferred over the mtDNA trees (Criterion 3; Table 2). The UCE results also make more sense than the mtDNA results when you consider that *A. picturata*—not *A. pulchella*—had been described as a member of the *Aprasia repens* species group on the basis of shared morphological characteristics and geography (Storr, Smith & Johnstone, 1990; Maryan, Bush & Adams, 2013). Given that the mtDNA pairing of *A. picturata* and *A. pulchella* appears to be spurious, this apparently anomalous pairing may represent another case of ancient mtDNA introgression. Evidence supporting this novel hypothesis comes from the observation that both species’ ranges are adjacent to each other (see maps in Storr, Smith & Johnstone, 1990 and Smith & Henry, 1999). If this hypothesis is correct, then it would explain why both UCE trees in *SB&O* and the allozyme tree in Maryan, Adams & Aplin (2015) did not show the same *A. pulchella*/*A. picturata* sister species pair recovered in the mtDNA tree.

Thus, the UCE and allozyme trees corroborate the hypothesis of *A. repens* group monophyly. It is notable that in all trees that contained members of the *A. repens* group, monophyly was only rejected in the mtDNA trees owing to an apparently anomalous sister species pairing between *A. pulchella* and *A. picturata*. However, as pointed out above, this unusual sister species relationship in the mtDNA trees may be an artifact of past hybridization. Thus, despite the minor topological conflict between mtDNA vs. all other trees, it seems likely that the traditional *A. repens* group is monophyletic as would be expected on morphological and biogeographical grounds.

### Phylogenetic relationships in the *Aprasia repens* species group

Having established that the *A. repens* group is probably monophyletic, we can now focus on the relationships among its eight members. Recall that in the two UCE trees (Fig. 11) *A. picturata* occupied the basal lineage in this group, whereas in the allozyme tree of Maryan, Adams & Aplin (2015) *A. smithi* was instead in this position with *A. picturata* as the second-most basal lineage (Fig. 12C). Thus, one commonality between the UCE and allozyme trees with respect to *A. picturata* is that in both cases this species was placed in a relatively basal position in the group. Which of the two placements for *A. picturata* is more probable? Because the UCE trees were inferred from far more characters than the allozyme tree, the placement of *A. picturata* in the UCE trees is preferred over the allozyme tree (Criterion 8; Table 2).

If we accept *A. picturata* as the basal lineage in the *A. repens* group, then we can attempt to find the most probable relationships among the remaining seven members of this clade given available evidence. Using allozyme data, Maryan, How & Adams (2013) inferred a phylogeny for five species of this group that showed their newly described species, *A. clairae*, as the sister species to *A. repens* (Fig. 12A). In a second allozyme-based study of this group, Maryan, Bush & Adams (2013) inferred a tree that not only showed a sister species relationship between *A. clairae* and *A. repens*, but also between *A. haroldi* and the newly described *A. litorea* (Fig. 12B). Maryan, Adams & Aplin (2015) described a third species, *A. wicherina*, and subsequently placed it as the sister species to *A. rostrata* in an allozyme phylogeny for all eight species of the *A. repens* complex (Fig. 12C). In all three allozyme trees *A. repens sensu stricto* was found to be the sister species to *A. clairae* (Figs. 12A–12C; Criterion 5; Table 2), while in two of the three trees the sister group to *A. repens*/*A. clairae* was a sister species group consisting of *A. haroldi* and *A. litorea* (Figs. 12B and 12C; Criterion 5; Table 2). Although the tree shown in Fig. 12A does not contain *A. litorea*, its topology is consistent with the other two trees (Figs. 12B and 12C). Because these two allozyme trees represent the only phylogenetic hypotheses containing *A. clairae*, *A. litorea*, and *A. haroldi*, we can accept the allozyme-suggested clade of (((*A. repens*, *A. clairae*), (*A. haroldi*, *A. litorea*))) as the current estimate of their relationships (Criterion 4; Table 2). Similarly, we can accept the sister species pairing of *A. rostrata* and *A. wicherina* in the tree inferred by *Maryan, Adams & How* (*2015*; Fig. 12C; Criterion 4; Table 2). We must now try to find the likely placements for the *A. rostrata*/*A. wicherina* lineage and *A. smithi* lineage within the *A. repens* species group.

Based on our earlier assessments, the *A. rostrata*/*A. wicherina* and *A. smithi* lineages appear to be located between the basal lineage of the *A. repens* species group (i.e., *A. picturata*) and the *A. repens*/*A. clairae*/*A. haroldi*/*A. litorea* clade. Assuming this to be true, then there are three possible topological arrangements involving *A. rostrata*/*A. wicherina* and *A. smithi* in relation to these other lineages: the first hypothesis holds that *A. smithi* is the sister lineage to the *A. repens*/*A. clairae*/*A. haroldi*/*A. litorea* clade; the second hypothesis suggests that *A. rostrata*/*A. wicherina* is the sister lineage to the *A. repens*/*A. clairae*/*A. haroldi*/*A. litorea* clade; and the third hypothesis holds that *A. rostrata*/*A. wicherina* and *A. smithi* could be sister lineages, and this lineage, in turn, is located between *A. picturata* and the *A. repens*/*A. clairae*/*A. haroldi*/*A. litorea* clade. Are any of these hypotheses favored by evidence?

All three mtDNA trees (Figs. 6A–6C), the concatenated UCE tree (Fig. 11B), and one of the allozyme trees (Fig. 12A) have topologies that support the first hypothesis. In contrast, two other allozyme trees (Figs. 12B and 12C) agree with the second hypothesis while the MSC-UCE tree (Fig. 11A) supports the third hypothesis. Although the allozyme tree in Fig. 12C contains all eight members of the *A. repens* group, the placement of *A. smithi* as the sister lineage to the rest of the group conflicts with its more nested position in all other trees that contained this species (Figs. 6, 11 and 12A). The placement of *A. smithi* in Fig. 12C therefore appears to be an error. Support for the first hypothesis is compelling when one considers that trees based on three independent datasets are in agreement with each other (Criterion 7; Table 2), though the lack of internal consistency among allozyme trees in this regard casts some doubt on this conclusion. With this caveat in mind, we can conclude that *A. smithi* is the probable sister lineage to the *A. repens*/*A. clairae*/*A. haroldi*/*A. litorea* clade. Synthesizing our conclusions about the *A. repens* species group, we can suggest a new phylogenetic hypothesis for *Aprasia* (Fig. 12D).

### Genus *Delma*

Kluge (1976) presented the first phylogenetic hypothesis for the genus *Delma*. However, that morphology-based study only included six of the 22 currently recognized species in *Delma* (Figs. 2 and 5). In subsequent DNA studies of pygopodid phylogeny, *JP*&*D* included 17 species of *Delma* (Figs. 3, 4, 6 and 7), *BB*&*J* sampled all 22 *Delma* species (Figs. 8 and 9), and *SB&O* included 14 species of *Delma* in their trees (Fig. 11). Thus *BB*&*J* conducted the only taxonomically complete phylogenetic study of *Delma* to date. We can use the four *Delma* species groups (i.e., Clades A, B, D, and Group C) that *BB*&*J* had defined as a framework for understanding *Delma* phylogeny (see Fig. 9), especially in light of the more recent phylogenomic study of pygopodids by *SB&O*.

### *Delma* Clade A

Both mtDNA and nDNA trees in *JP*&*D* independently suggested that *D. australis* AG Kluge was the sister species to *D. torquata* AG Kluge (Figs. 6 and 7) thus providing strong evidence for this two-species group (Criterion 6; Table 2). Later, Maryan et al. (2015) conducted a morphological and molecular investigation of the geographically variable *D. australis*. Interestingly, their mtDNA, nDNA, and concatenated mtDNA + nDNA trees all showed *D. australis* to be paraphyletic—some populations of *D. australis* were found to be the sister group to *D. torquata* while another population of *D. australis*, isolated in southern Western Australia, was the sister lineage to a *D. australis*/*D. torquata* clade (see Fig. 1 in Maryan et al., 2015). To reconcile the taxonomy of *D. australis* with their phylogenetic results, Maryan et al. (2015) described the southern Western Australia population of *D. australis* as a new species, *Delma hebesa* B Maryan, IG Brennan, M Adams, & KP Aplin.

On morphological grounds, *D. australis*, *D. torquata*, and *D. hebesa* form a presumably monophyletic species group (Maryan et al., 2015). Indeed, the studies by *JP*&*D* and Maryan et al. (2015) provided molecular phylogenetic evidence that supports this hypothesis. However, as pointed out earlier, adequate taxon sampling is a requirement for robust tests of species group monophyly hypotheses. Looking again at the taxon sampling in these studies, we see that *JP*&*D* sampled most species of *Delma* but did not include *D. hebesa* while the Maryan et al. (2015) study did not include most species of *Delma* in their trees. In the latter study, these workers used two species of *Delma* as outgroups to root an ingroup consisting of *D. australis*, *D. torquata*, and *D. hebesa*. Thus, their taxonomic sampling scheme implies that monophyly of these three species had been assumed a priori rather than a hypothesis to be tested using the trees themselves. We can perform a partial test of the group’s monophyly using an unrooted version of the tree in Maryan et al. (2015); when we do this we can see that a single branch separates the outgroup cluster from the ingroup cluster, an observation that permits us to conclude that the unrooted tree is evidence consistent with ingroup monophyly. In a more comprehensive study of *Delma* phylogeny by *BB*&*J*, the authors included all species of *Delma* in their mtDNA and nDNA trees as well as many other outgroup species from other genera. Thus, their results (Fig. 8) provided the best possible evidence for the monophyly of the ((*D. australis*, *D. torquata*), *D. hebesa*) group that can be obtained from a single tree. And because this monophyletic group was recovered in two independent trees (mtDNA and nDNA) in *BB*&*J*, we can conclude that the hypothesis by Maryan et al. (2015) has been independently corroborated (Criterion 6; Table 2). Given the strength of their results, *BB*&*J* defined this three-species group as “Clade A” (Fig. 9).

### *Delma* Clade B

Kluge (1974) described the species *Delma nasuta* A.G. Kluge, but this “species” was later revealed to be a cryptic species group when Storr (1987) split *D. nasuta* into three species on the basis of morphological differences: *D. nasuta sensu stricto*, *D. butleri* G.M. Storr, and *D. haroldi* G.M. Storr. Mitochondrial DNA tree results in *JP*&*D* and in *BB*&*J* suggested that this species group was monophyletic with *D. butleri* and *D. haroldi* appearing to be sister species (Figs. 6 and 8A). Surprisingly, however, the nDNA trees in *BB*&*J* (Fig. 8B) placed *D. grayii*—not *D. nasuta*—as the sister lineage to the *D. butleri*/*D. haroldi* species pair, thus contradicting the mtDNA results. Another interesting difference between mtDNA and nDNA hypotheses of *Delma* relationships concerns the placement of *D. inornata* A.G. Kluge. In mtDNA trees, this species was placed with *D. fraseri*, *D. grayii*, and *D. petersoni* GM Shea (Figs. 6 and 8A). In contrast, evidence from nDNA trees in *BB*&*J* suggested that *D. inornata* was the sister lineage to the clade containing *D. butleri*, *D. haroldi*, and *D. grayii* with *D. nasuta* pushed even further outside as the sister lineage to the other four species (Fig. 8B).

As mentioned earlier, *BB*&*J* hypothesized that the apparent sister species relationship between *D. grayii* and *D. fraseri* in mtDNA trees was due to historical introgression between those two species rather than by common ancestry. They argued that their nDNA trees exhibited the more probable placement for *D. grayii*; that is, nested in a clade containing *D. butleri*, *D. haroldi*, *D. inornata*, and *D. nasuta* (Fig. 8B). Additional significant support for their hypothesis came from the study of *SB&O*, as their MSC-UCE tree showed a monophyletic group comprised of *D. butleri*, *D. haroldi*, *D. grayii*, and *D. nasuta* (Fig. 11A). Unfortunately, their tree did not include *D. inornata* and the relationships among the *D. butleri*/*D. haroldi*, *D. grayii*, and *D. nasuta* lineages were not resolved.

**Figure 13:**
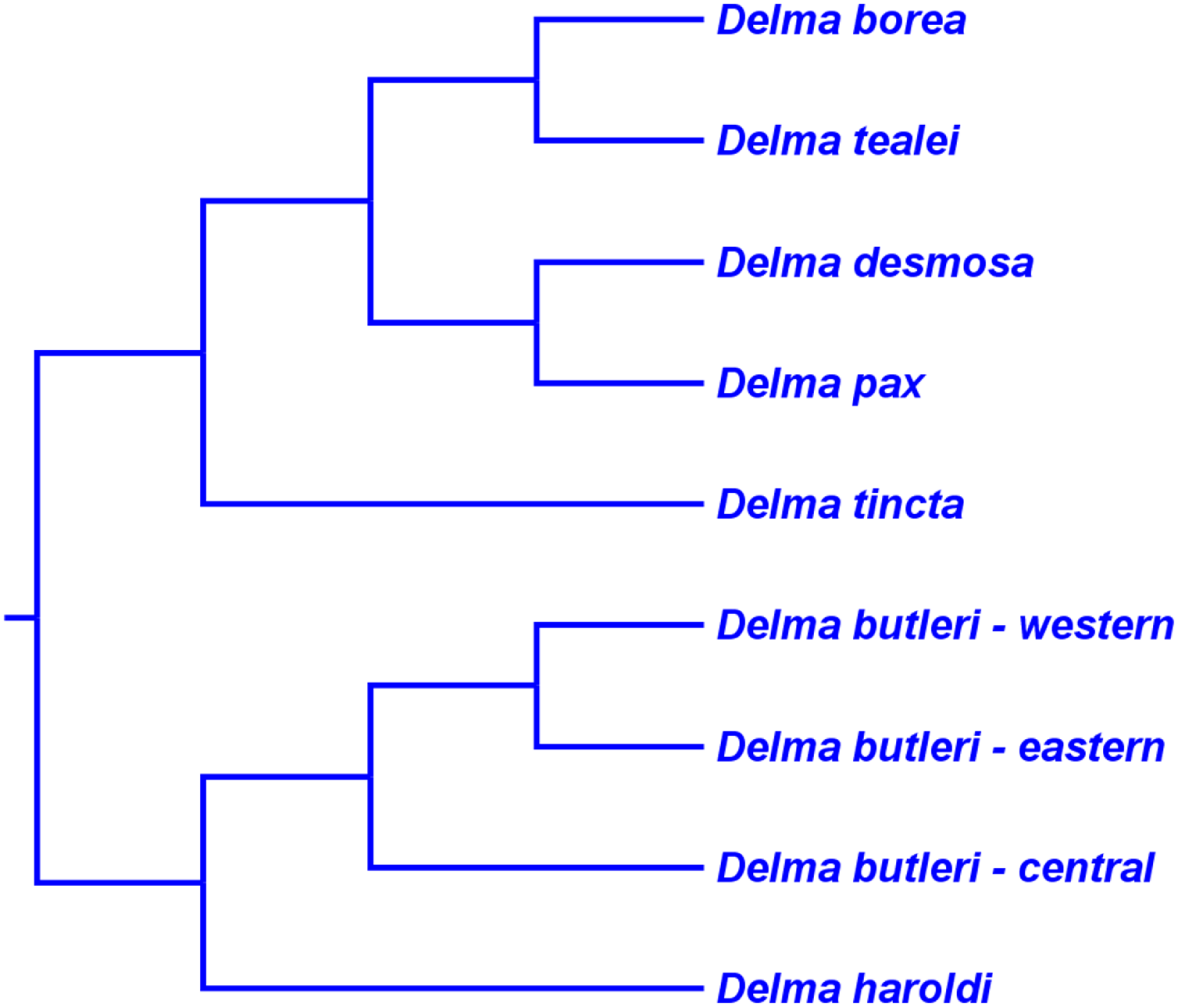
Phylogenetic hypothesis of the *Delma tincta* group based on allozyme data. Cladogram of the inferred relationships among the five species belonging to the *D. tincta* group. Conspecific species *D. butleri* (several geographical populations sampled) and *D. haroldi* were included because they were believed to be closely related to members of the *D. tincta* group. Tree was rooted using the midpoint method. After Fig. 5 in Maryan, Aplin & Adams (2007).

Surprisingly, the concatenated UCE tree in *SB&O* showed novel—but likely spurious—sister species relationships between *D. butleri and D. tincta*, and between *D. haroldi* and *D. borea* (Fig. 11B). These two highly unexpected sister species pairs are probably erroneous for several reasons. First, on morphological grounds *D. butleri* and *D. haroldi* are so similar to each other that they were formerly considered to be the same species along with *D. nasuta* (Kluge, 1974; Storr, 1987; Shea, 1991). Similarly, *D. borea* and *D. tincta* have long been recognized as close relatives in the *Delma tincta* species group (see below). Multiple independent molecular phylogenies also refute these two suspect sister species pairs (see Figs. 6, 8, 11A and 13). Therefore, monophyly of the *D. butleri*/*D. haroldi* sister species pair is well supported by independent datasets (Criterion 6; Table 2). We can also see that two independent MSC trees (Figs. 8B and 11A) support this sister species pair over the alternative topology found in the concatenated UCE tree (Fig. 11B; Criterion 3; Table 2). The relationships among *D. butleri*, *D. haroldi*, *D. grayii*, and *D. nasuta* observed in the MSC-UCE tree in *SB&O* are consistent with nDNA trees in *BB*&*J* and thus provide strong evidence that these four species are each other’s closest living relatives if *D. inornata* is ignored.

As we have seen, there are two hypothesized placements for *D. inornata*: as the sister lineage to *D. fraseri*/*D. petersoni* in mtDNA trees (Figs. 6 and 8A) or in a clade with *D. butleri*, *D. haroldi*, *D. grayii*, and *D. nasuta* in nDNA trees (Fig. 8B). Which of these placements is more likely correct? Because the nDNA dataset in *BB*&*J* was larger than the mtDNA datasets, I regard the nDNA placement as the best evidence supporting the placement of *D. inornata* (Criterion 8; Table 2).

The nDNA trees in *BB*&*J* (Fig. 8B) provide the only evidence yet for the phylogenetic relationships among *D. butleri*, *D. haroldi*, *D. grayii*, *D. inornata*, and *D. nasuta*. The *D. butleri*/*D. haroldi* sister species group is now well established but the remaining relationships are still questionable. In particular, the relative placements of *D. grayii* and *D. nasuta* in those trees are counterintuitive as the latter species would be expected to be the sister lineage to the *D. butleri*/*D. haroldi* lineage on morphological grounds instead of *D. grayii* (Storr, 1987). Despite the analysis of thousands of UCE loci, it is striking that the MSC-UCE tree was not able to show the likely sister lineage (i.e., *D. grayii* or *D. nasuta*) to the *D. butleri*/*D. haroldi* clade. It was also surprising that the concatenated UCE loci yielded a tree containing likely spurious sister species pairings of *D. butleri*/*D. tincta* and *D. haroldi*/*D. borea*. But given that the members of this five-species group (except *D. grayii*) in addition to *D. borea* and *D. tincta* have overlapping or proximal contemporary geographic ranges (Storr, Smith & Johnstone, 1990), the possibility exists that ancient hybridization involving these lineages is clouding their true phylogenetic relationships in molecular studies (e.g., Lásková, Landová & Frynta, 2015; Zhang et al., 2021).

In summary, evidence from two independent trees (Figs. 8B and 11A) points to a clade comprised of at least *D. butleri*, *D. haroldi*, *D. grayii*, and *D. nasuta*. One nDNA tree (Fig. 8B) suggests that *D. inornata* is the fifth member of this clade though this must be independently corroborated in a future study. Morphological evidence supporting this five-species group also exists, as *BB*&*J* stated that these species are unique among species of *Delma* in that they lack dark-colored neck bands, which contrasts with the more common banded-neck condition found in many species of *Delma* such as *D. fraseri* and *D. petersoni*. Accordingly, *BB*&*J* named the five-species clade that contained *D. butleri*, *D. haroldi*, *D. grayii*, *D. inornata*, and *D. nasuta* in their nDNA trees “Clade B” (Fig. 9).

### *Delma* Group C

Brennan, Bauer & Jackman (2016) defined this five-species group to include *D. impar* JG Fischer, *D. molleri* C Lütken, *D. plebeia*, *D. fraseri*, and *D. petersoni* (Fig. 9). Unlike Clades A, B, and D, Group C is not a monophyletic group (Fig. 9). We will begin our review of this group by focusing on *D. impar*, *D. molleri*, and *D. plebeia* before considering the evidence supporting the placements of *D. fraseri* and *D. petersoni*.

The morphology tree of *K76* showed *D. impar* and *D. molleri* as being sister species (Fig. 5). This sister species pair has since been recovered in *JP*&*D*’s and *BB*&*J*’s mtDNA trees (Figs. 6 and 8A, respectively) and in *BB*&*J*’s nDNA trees (Fig. 8B) thereby making this an independently corroborated clade (Criterion 6; Table 2). The mtDNA trees in *BB*&*J* exhibited *D. mitella* as the sister species to *D. plebeia*, and this lineage was, in turn, the sister lineage to the *D. impar*/*D. molleri* pair (Fig. 8A). The mtDNA trees of *JP*&*D* did not include *D. plebeia*, but they did consistently show *D. mitella* as the sister lineage to the *D. impar*/*D. molleri* clade (Fig. 6), which was consistent with the mtDNA results in *BB*&*J*. In contrast, the nDNA trees in *BB*&*J* only placed *D. plebeia* as the sister lineage to *D. impar*/*D. molleri* lineage, while *D. mitella* was instead located as a long single-species lineage deeper in the *Delma* clade (Fig. 8B). Although the nDNA trees of *JP*&*D* did not include *D. plebeia*, they did place *D. mitella* in the same position of the tree as was later found in the nDNA trees in *BB*&*J* (compare the placements of *D. mitella* in Figs. 7 and 8B). The placement of *D. mitella* as the sister lineage to the *D. impar* and *D. molleri* clade in all mtDNA trees demonstrates that this part of the mtDNA gene tree has likely been accurately reconstructed. Likewise, the placement of *D. mitella* deeper in the *Delma* tree by several independent nuclear genes provides convincing evidence that the nDNA trees were also accurately reconstructed with respect to *D. mitella*. Given these discordant results between mtDNA and nDNA trees, *BB*&*J* argued that ancient mtDNA introgression between *D. mitella* and *D. plebeia* best explained the placement of *D. mitella* in the mtDNA trees. If we therefore attribute the placement of *D. mitella* in the mtDNA trees in *BB*&*J* as an artifact of past introgression, then both mtDNA and nDNA agree that *D. plebeia* is the sister lineage to the *D. impar*/*D. molleri* lineage (Fig. 8). Because *D. mitella* is not a member of Group C, we will defer our discussion of its placement in the *Delma* phylogeny.

We now focus on the sister species pairing between *D. fraseri* and *D. petersoni*, and the placement of this lineage in Group C (Fig. 9). The mtDNA trees of *JP*&*D* and *BB*&*J* showed *D. fraseri* as the sister species to *D. grayii*, with *D. petersoni* occupying the sister position to this hypothesized sister species pair. Brennan, Bauer & Jackman (2016) obtained evidence showing that this surprising result must have been due to historical mtDNA introgression between *D. fraseri* and *D. grayii*. If true, then this implies that the allopatric *D. fraseri* and *D. petersoni* are sister species—a logical result given that both were originally subspecies of *D. fraseri* (Shea, 1991). Indeed, the sister species relationship between *D. fraseri* and *D. petersoni* has now been strongly supported by the nDNA results in *BB*&*J* (Figs. 8B and 9) and both UCE trees in *SB&O* (Fig. 11*)*, thereby adding support to the mtDNA introgression hypothesis of *BB*&*J* and leaving little doubt that *D. fraseri* and *D. petersoni* are sister species (Criterion 6; Table 2).

From our earlier analysis of Clade B, we saw that strong evidence exists supporting the placement of *D. grayii* among the members of that group. Thus, if we ignore the placement of *D. grayii* in the mtDNA trees and assume the other relationships are accurate, notice that *D. fraseri*/*D. petersoni* lineage forms a monophyletic group with the five members of Clade B (Figs. 6 and 8A). Interestingly, the MSC-UCE tree of *SB&O* recovered this same clade except that *D. inornata* was missing from that tree (Fig. 11A). In contrast, the concatenated nDNA trees in *BB*&*J* placed the *D. fraseri*/*D. petersoni* lineage in a clade comprised of *D. impar*, *D. molleri*, *D. plebeia*, *D. labialis, D. elegans* AG Kluge, *D. tincta*, *D. borea*, *D. tealei*, *D. pax* AG Kluge, and *D. desmosa* B Maryan, KP Aplin & M Adams—a clade they subsequently split up into Group C, Clade D, and a single-species lineage, *D. labialis* (Figs. 8B and 9).

Given that both mtDNA and the UCE trees independently agree that the *D. fraseri*/*D. petersoni* lineage is the sister lineage to Clade B, it appears that the concatenated nDNA tree of *BB*&*J* shows an incorrect placement for these two species, possibly due to long-branch attraction (Swofford et al., 1996) or ancestral polymorphisms (Maddison, 1997). Regarding the latter possibility, although *BB*&*J* did use an MSC-based species tree method, their dataset only consisted of three presumably independent nDNA loci. This sample size of independent loci is too low to generate an adequate empirical distribution of reconstructed gene trees and thus the reliability of their species tree estimate must be low.

In summary, compelling evidence exists supporting the placement of the *D. fraseri*/*D. petersoni* lineage with members of Clade B (Criteria 3 and 8; Table 2). If we accept this rearrangement, then species membership for Clade B and Group C must be revised. Accordingly, I here define “Clade B2,” to contain *D. nasuta*/*D. butleri*/*D. haroldi*/*D. grayii*/*D. inornata*/*D. fraseri*/*D. petersoni*, and “Group C2,” now a monophyletic group, to include *D. impar*/*D. molleri*/*D. plebeia*.

### *Delma* Clade D

This clade includes the five-species *Delma tincta* group plus a single-species lineage, *D. elegans* (Fig. 9). We will first review the evidence supporting monophyly of the *D. tincta* group before delving into the phylogenetic relationships among its species. We will then examine the evidence supporting placement of *D. elegans* as the sister lineage to the *D. tincta* group.

### Testing monophyly of the *Delma tincta* species group

Based on morphological characters, Shea (1991) defined the *D. tincta* group to include *D. borea*, *D. tincta*, and *D. pax*. Later, *JP*&*D* obtained strong evidence supporting monophyly of the group in their mtDNA (Fig. 6) and nDNA (Fig. 7) trees (Criterion 6; Table 2). In a subsequent taxonomic and phylogenetic study of the this group, Maryan, Aplin & Adams (2007) added two new species—*D. desmosa*, and *D. tealei*—to this group, thus increasing group membership to five species. In their phylogenetic hypothesis, which was based on allozyme data, these authors not only included individuals from all five members of this presumed monophyletic group, but they also included samples from two closely related species, *D. butleri* and *D. haroldi* (Fig. 13). Although the inclusion of species outside the presumed species group (i.e., outgroup) would typically be used to root the tree, the authors of this study instead rooted their tree using the midpoint method. Thus, their taxon-sampling scheme and tree-rooting approach permited a valid test of the *D. tincta* group monophyly hypothesis. Looking at the allozyme tree in Maryan, Aplin & Adams (2007; Fig. 13), we see that this group is monophyletic as expected. These results provided the first evidence supporting monophly of the five-species *D. tincta* group, though inclusion of additional species of *Delma* would have further strengthened their analysis. Brennan, Bauer & Jackman (2016) obtained results strongly supporting group monophyly, as their mtDNA (Fig. 8A) and nDNA (Fig. 8B) trees showed a monophyletic *D. tincta* group in a clade containing all other species of *Delma* (Criterion 6; Table 2). The MSC-UCE tree (Fig. 11A) in *SB&O* also showed all five *D. tincta* group species as a monophyletic group that was nested among nine other species of *Delma*. Given that the five-member *D. tincta* group was found to be monophyletic in four independent trees, we can conclude that monophyly of this species group is independently corroborated (Criterion 6; Table 2).

### Phylogenetic relationships inside the *Delma tincta* species group

We now turn our attention to the relationships among the species inside the *D. tincta* group. The allozyme tree in Maryan, Aplin & Adams (2007) suggested a sister species relationship between *D. pax* and *D. desmosa*, and a sister species pairing between *D. borea* and *D. tealei*, while *D. tincta* was placed as the sister lineage to both two-species clades (Fig. 13). The mtDNA and nDNA trees in *BB*&*J* also showed a *D. pax* and *D. desmosa* sister species pair, but the other sister species pair in those trees was comprised of *D. tincta* and *D. tealei* (Fig. 8). However, the placement of *D. borea* varied between their mtDNA and nDNA trees: in the mtDNA trees, *D. borea* was the sister lineage to the *D. tincta*/*D. tealei* pair (Fig. 8A), while *D. borea* was the sister lineage to the other four species in their nDNA trees (Fig. 8B). Based on the results of phylogenetic hypothesis testing, *BB*&*J* ruled out the possibility that the conflicting placements of *D. borea* could be attributed to weak phylogenetic signal in their nDNA dataset. This finding, along with the knowledge that their nDNA dataset was larger than their mtDNA dataset, evidently prompted *BB*&*J* to cast doubt on their mtDNA results concerning the placement of *D. borea*. They argued that ancient mitochondrial introgression between *D. borea* and the most recent common ancestor to the *D. tincta*/*D. tealei* sister species pair created an mtDNA tree that did not reflect the true speciational history for this group. Accordingly, they favored the placement of *D. borea* in their nDNA trees. Skipwith, Bi & Oliver (2019) also inferred the phylogenetic relationships among all five members of the *D. tincta* group. In both their MSC- and concatenated UCE trees, they recovered the same *D. pax* and *D. desmosa* sister species pair (Fig. 11). However, in their MSC-UCE tree *D. borea* and *D. tincta* were grouped together as sister species, while *D. tealei* was the sister lineage to the *D. pax*/*D. desmosa* clade (Fig. 11A)—thereby yielding a fourth unique tree hypothesis for the *D. tincta* group. With the exception of the *D. pax*/*D. desmosa* pair, the other *D. tincta* group relationships in the concatenated UCE tree are likely in error (Fig. 11B), as previously discussed, and thus will not be considered further.

All tree hypotheses that contained all members of the *D. tincta* group showed a *D. pax* and *D. desmosa* sister species pair. This evidence therefore constitutes strong support for monophyly of these two species (Criterion 6; Table 2). However, we are still left with four different independent tree hypotheses to consider involving the relationships among *D. borea*, *D. tincta*, and *D. tealei*. Several possible reasons for these incongruencies exist.

Given that *D. tincta* and *D. tealei* were found to be sister species in *BB*&*J*’s mtDNA (Fig. 8A) and nDNA (Fig. 8B) trees, they hypothesized that past introgression between *D. borea* and the ancestral species to *D. tincta*/*D. tealei* could explain their presumably erroneous mtDNA results regarding the placement of *D. borea*. Although the *D. tincta*/*D. tealei* pair was recovered in the concatenated mtDNA + nDNA tree in *B&O* (Fig. 10), this tree was largely based on the same nDNA sequences (i.e., *C-mos*, *DYNLL1*, and *RAG1* genes) used by *BB*&*J* and thus there is a lack of independence between these tree hypotheses. Another limitation of the *B&O* tree is that it did not include mtDNA sequences for *D. borea*. Thus, the placement of *D. borea* in *B&O*’s tree must be solely due to the nDNA signal in their dataset and indeed it matches the placement found in *BB*&*J*’s nDNA trees (Fig. 8B).

In phylogenetic hypotheses of the *D. tincta* group based on UCE and allozyme datasets, *D. tincta* and *D. tealei* were non-sister species (Figs. 11A and 13). If *D. tincta* and *D. tealei* are in reality non-sister species, then there are at least two historical scenarios that could cause them to be incorrectly grouped together in DNA-based trees. One possibility is that the recovered *D. tincta* and *D. tealei* sister pairs in trees could be the result of ancestral polymorphisms being present in either or both the mtDNA and nDNA data (Maddison, 1997). Such as scenario is plausible in this case because, as *BB*&*J* argued, the *D. tincta* group appears to represent a recent and rapid species radiation. Short time intervals between speciation events can lead to the existence of ancestral polymorphisms in contemporary populations or species, which, in turn, can cause gene tree - species tree conflicts *(Wakeley, 2009)*. Although *BB*&*J* inferred a species tree using the MSC approach, their sample size of three presumably independent loci was likely too low to provide a reliable tree estimate. Another historical scenario is that past hybridization between *D. tincta* and *D. tealei* could cause these two species to appear as sister species in trees when instead they are non-sister species. This scenario is plausible given that *D. tincta* and *D. tealei* are sympatric with each other (Maryan, Aplin & Adams, 2007).

However, as the recent study by Zhang et al. (2021) showed, extensive ancient gene flow between non-sister species can confound species tree estimates based on genome-wide datasets but perhaps not negatively impact tree estimates inferred from smaller numbers of genomic loci. This finding raises the possibility that the *D. borea*/*D. tincta* sister species pair in the MSC-UCE tree may actually be an artifact of hybridization, a plausible scenario given that both of these species are sympatric with each other (Storr, Smith & Johnstone, 1990).

In summary, there is no doubt about the sister species pairing of *D. pax* and *D. desmosa*, but the remaining relationships within the *D. tincta* group involving *D. borea*, *D. tincta*, and *D. tealei* are still messy owing to the existence of four different independent tree hypotheses. A key unresolved issue is whether *D. tincta* and *D. tealei* are sister or non-sister species. Because two independent trees support the former hypothesis (mtDNA and nDNA trees in *BB*&*J*; Fig. 8) and two independent trees support the latter (MSC-UCE and allozyme trees in *SB&O* and *Maryan et al., 2007*, respectively; Figs. 11A and 13), neither hypothesis can be outright favored on a majority-rule basis. Thus, choosing a preferred tree hypothesis is proving to be especially difficult. I am inclined to favor the MSC-UCE tree hypothesis for this group because it alone was based on a large number of presumably independent loci that were analyzed in an MSC framework (Criterion 3; Table 2).

### Phylogenetic placement of *Delma elegans* in relation to the *D. tincta* group

The mtDNA and nDNA trees in *BB*&*J* placed *D. elegans* as the sister lineage to the *D. tincta* group (Fig. 8). In contrast, the concatenated mtDNA + nDNA tree in *B&O* showed

*D. elegans* and *D. labialis* to be a weakly-supported (i.e., low BPP node support) sister species pair, which, in turn, was the sister group to the *D. tincta* group (Fig. 10). The UCE trees in *SB&O* showed an alternative arrangement, as they placed *D. impar* as the sister lineage to the *D. tincta* group, with *D. elegans* placed outside as the sister lineage to a *D. impar* + *D. tincta* group clade (Fig. 11). Although the placement of *D. elegans* suggested by the UCE trees can be favored over the those suggested by mtDNA and nDNA trees in *BB*&*J* on the basis of Criterion 8, Criteria 2 and 6 favor the alternative hypothesis suggested by the mtDNA and nDNA trees in *BB*&*J* (Table 2). Criterion 2 can be invoked because the UCE trees lacked two of the three Group C2 members, thus leaving *D. impar* more vulnerable to being long-branch attracted to the *D. tincta* group clade given the short internodes separating Group C2 species from *D. elegans* (see Fig. 2 in *BB*&*J*). Accordingly, the *D. tincta* group + *D. elegans* clade appears to have more substantial support than the alternative topology found in the UCE trees. Also, given the geographical distributions, it is more plausible that *D. elegans* would be phylogenetically closer to the *D. tincta* group than to any member of Group C2. This is because the *D. tincta* group and *D. elegans* are all found in the deserts of northwestern Australia, while Group C2 members are restricted to a temperate region in southeastern Australia—these regions being separated by thousands of kilometers of desert.

### *Delma* phylogeny: a new hypothesis

The four major groups of *Delma* defined by *BB*&*J* account for 19 of the 22 recognized species in the genus. Based on conflicting evidence from other studies, I suggested a revision of two of those groups. Specifically, I recommended transferring the *D. fraseri*/*D. petersoni* sister species pair from Group C to Clade B, which resulted in two revised groups—Clade B2 and Group C2. Table 3 lists the members belonging to these groups. We will now consider the phylogenetic relationships among these four groups before we evaluate the hypothesized placements of the three highly divergent species of *Delma* (i.e., *D. (Aclys) concinna*, *D. labialis*, and *D. mitella*) in the *Delma* tree.

**Table 3:** Major species groups in the pygopodid genus *Delma*.

Group	Species of *Delma* assigned to each clade or group	Source
Clade A	*australis* + *torquata + hebesa*	Brennan, Bauer & Jackman (2016)
Clade B	*nasuta* + *inornata* + *grayii + butleri* + *haroldi*	Brennan, Bauer & Jackman (2016)
Clade B2	*nasuta* + *inornata* + *grayii* + *butleri + haroldi +* ***fraseri* + *petersoni***	This study
Group C	*impar* + *molleri* + *plebeia* + ***fraseri*** + ***petersoni***	Brennan, Bauer & Jackman (2016)
Group C2	*impar* + *molleri* + *plebeia*	This study
Clade D	*tincta + tealei* + *borea* + *pax* + *desmosa + elegans*	Brennan, Bauer & Jackman (2016)

**Note:**

These species comprise 19 of 22 species in *Delma*. Three single-species lineages (*D. concinna*, *D. labialis*, *D. mitella*) are not closely allied with any of these groups. Note that the sister species pair *D. fraseri* and *D. petersoni* (in bold) was originally included in Group C but more recent phylogenetic evidence suggests that this species pair instead belongs with members of Clade B (see main text).

**Figure 14:**
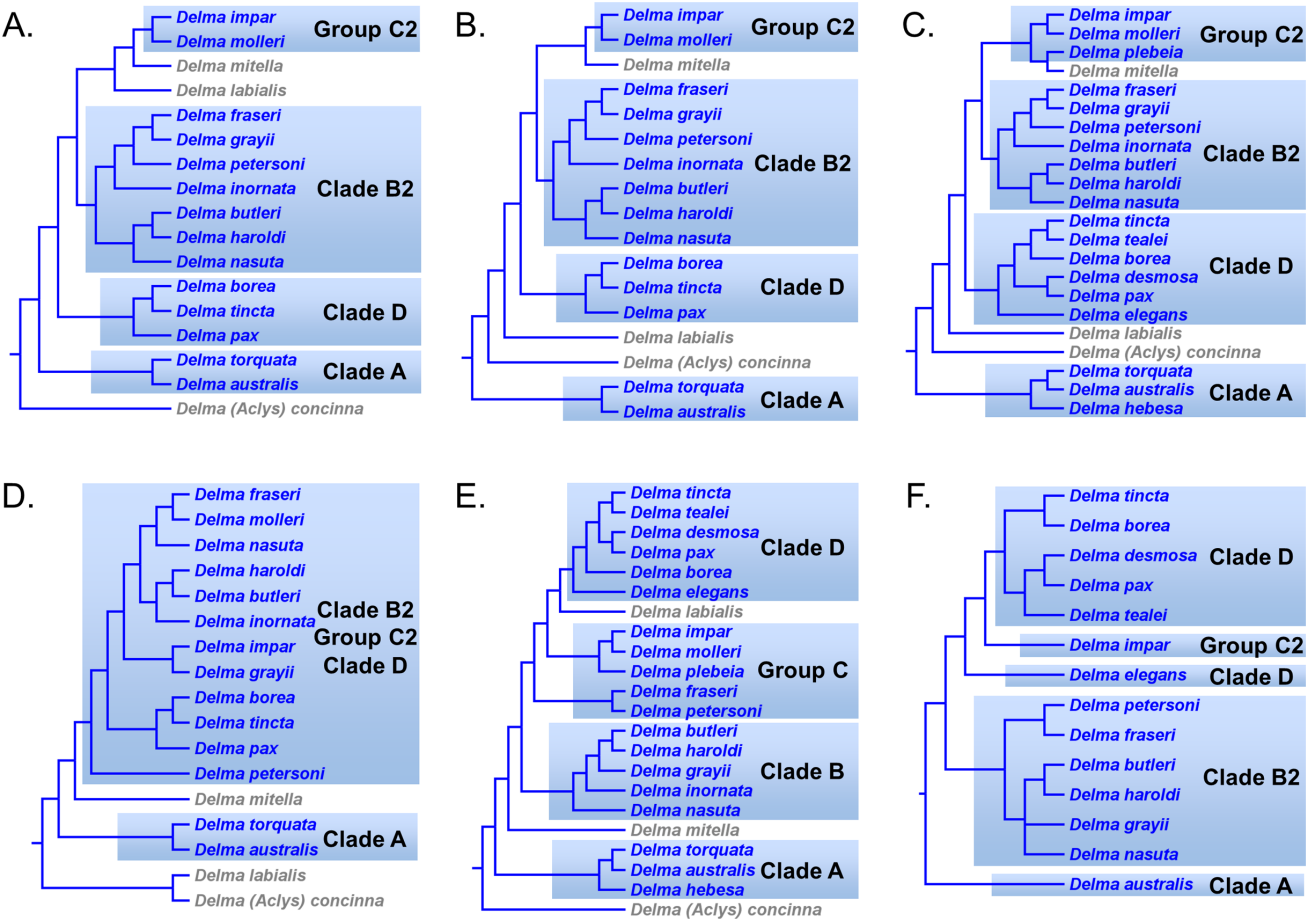
Hypothesized relationships among the four main *Delma* species groups. (A) Maximum parsimony mtDNA tree based on Fig. 6A (modified version of Fig. 2A in Jennings, Pianka & Donnellan, 2003). (B) Maximum likelihood/Bayesian inference mtDNA tree based on Figs. 6B and 6C (modified versions of Figs. 2B and 2C in Jennings, Pianka & Donnellan, 2003). (C) Maximum likelihood/Bayesian inference mtDNA tree based on Fig. 8A (modified version of Fig. 1 in Brennan, Bauer & Jackman, 2016). (D) Maximum parsimony/maximum likelihood/Bayesian inference nDNA tree based on Fig. 7 (modified version of Fig. 4 in Jennings, Pianka & Donnellan, 2003). (E) Maximum likelihood/Bayesian inference nDNA tree based on Fig. 8B (modified version of Fig. 1 in Brennan, Bauer & Jackman, 2016). (F) Multispecies coalescent (MSC) UCE species tree based on Fig. 11A (modified version of Fig. 4 in Skipwith, Bi & Oliver, 2019). Species names in blue belong to one of the four main species groups (each shaded in blue). See Table 3 for a list of species in each group. Species names in gray represent divergent single-species lineages not closely allied with any group.

If we examine each of the published phylogenetic hypotheses of the *Delma* clade with a focus on the placements of Clade A, Clade B2, Group C2, and Clade D, then one obvious generality that emerges is that Clade A exclusively occupies the basal position in each tree (Fig. 14). This is true for both mtDNA (Figs. 14A–14C) and nDNA (Figs. 14D–14F) trees and so this placement is independently corroborated (Criterion 7; Table 2). However, the relative positions of the other three groups vary between mtDNA and nDNA trees. If we ignore *D. labialis* and *D. mitella* in the three mtDNA trees, then Clade B2 and Group C2 are sister groups to each other with Clade D sitting as their sister group (Figs. 14A–14C).

The *Delma* clade within the nDNA tree in *JP*&*D* shows a largely unresolved superclade comprised of members of Clade B (or B2), Group C (or C2), and Clade D (Fig. 14D). Indeed, with the exceptions of the *D. butleri*/*D. haroldi* sister species pair, and the clade comprised of *D. borea*/*D. tincta*/*D. pax*, the remaining relationships within this superclade are likely wrong owing to a lack of phylogenetic signal from the highly conserved *C-mos* gene sequences upon which the tree was based. Thus, their nDNA tree is not informative about the relationships amongst these other three *Delma* groups.

However, nDNA trees inferred in other studies did show well-resolved *Delma* clades. In *BB*&*J* and *SB&O*, Clade B (or B2)—not Clade D—was placed as the sister group to the remaining two groups (Figs. 14E and 14F). This result remains robust regardless whether the phylogenetic location of the *D. fraseri*/*D. petersoni* sister species pair is found in Group C (Fig. 14E) or in Clade B2 (Fig. 14F). If we ignore *D. labialis* and the *D. fraseri*/*D. petersoni* sister species pair, then the remainder of Group C is the sister group to Clade D in *BB*&*J*’s nDNA tree (Fig. 14E). This same pattern can also be seen in *SB&O’s* nDNA tree except that only one member of Group C was sampled (*D. impar*), and it was located as the sister lineage to the *D. tincta* group (Fig. 14F). However, as I argued earlier, placement of *D. elegans* in the UCE trees in *SB&O* was likely an error and that this species is probably closer to Clade D than are any members of Group C (or C2).

The agreement among mtDNA trees concerning the relationships among Clade B (or B2), Group C (or C2), and Clade D is not surprising given that all mtDNA sites are linked and thus all have the same gene tree for any given set of species (Moore, 1995). In contrast, the general agreement between the nDNA trees in *BB*&*J* and *SB&O* regarding the alternative topology for these same groups is more compelling for two reasons. First, the datasets from each study contained genes that have independent genealogical histories yet they show substantial agreement between the inferred trees (Criterion 7; Table 2). Second, the MSC-UCE tree in *SB&O* was based on thousands of presumably independent loci, which were analyzed in an MSC framework (Criteria 3, 7, and 8; Table 2). I therefore accept the topological pattern of these four main groups suggested by the nDNA trees as being preferable over the mtDNA topology. Accordingly, I conclude that the evidence best supports a *Delma* tree with the following rooted topology for the four groups: ((((Group C2, Clade D), Clade B2), Clade A)).

We now consider the phylogenetic placements of *D. (Aclys) concinna*, *D. labialis*, and *D. mitella* relative to the four major groups in the *Delma* tree. In studying the various *Delma* tree hypotheses, it is evident that *D. (Aclys) concinna* probably occupies a basal or near-basal position in the tree (Figs. 14A–14E). In the MP mtDNA (Fig. 14A) and nDNA (Fig. 14D) trees in *JP*&*D*, and in the nDNA tree in *BB*&*J* (Fig. 14E), this species was either the sole member or a sister member (with *D. labialis*) of the basal lineage in the genus. The ML and BI mtDNA trees in *JP*&*D* (Fig. 14B) and in *BB*&*J* (Fig. 14C) showed Clade A occupying the basal position in the *Delma* tree with *D. (Aclys) concinna* located as the sister lineage to a clade comprising all remaining *Delma* species. Given the agreement among trees shown in Figs. 14A, 14D, and 14E, the evidence best supports the hypothesis that *D. (Aclys) concinna* alone occupies the basal lineage to the genus (Criterion 7; Table 2), though the ML and BI mtDNA trees contradict this conclusion (Figs. 14B and 14C).

Identifying the correct placement for *D. labialis* in the *Delma* tree is more problematic given the various hypothesized locations for this species (Figs. 14A–14E). The MP mtDNA tree in *JP*&*D* placed this species as the sister lineage to a clade containing *D. mitella* and Group C2 (Fig. 14A), while in the ML and BI mtDNA trees in *JP*&*D* (Fig. 14B) and in *BB*&*J* (Fig. 14C) *D. labialis* was the sister lineage to a clade comprised of Clade B2, Group C2, and Clade D. The nDNA tree in *JP*&*D* placed *D. labialis* as the sister species to *D. (Aclys) concinna* at the base of the *Delma* tree (Fig. 14D), while the nDNA tree in *BB*&*J* had positioned this species as the sister lineage to Clade D (Fig. 14E). On the basis of Criterion 8 (Table 2), I prefer the Clade D + *D. labialis* arrangement in the nDNA tree in *BB*&*J* because their dataset contained an order of magnitude more sites (3,019 bp) than the nDNA dataset (373 bp) used in *JP*&*D*.

Two different phylogenetic placements have been suggested for *D. mitella*. In all mtDNA tree hypotheses, *D. mitella* was consistently placed with members of Group C2 (Figs. 14A–14C), which differed from the deeper placement in the *Delma* clade suggested by two nDNA trees (Figs. 14D and 14E). Brennan, Bauer & Jackman (2016) argued that the mtDNA placement of *D. mitella* was likely spurious owing to ancient mitochondrial introgression between this species and an ancestor in the *D. plebeia* lineage. These authors obtained support for this hypothesis from the results of phylogenetic hypothesis testing, which suggested that their nDNA result was not due to weak phylogenetic signal. The nDNA trees in *JP*&*D* and *BB*&*J* placed *D. mitella* as the sister lineage to a clade containing Clade B (or B2), Group C (or C2), and Clade D (Figs. 14D and 14E). The agreement between these nDNA trees regarding the placement of *D. mitella* is compelling. It should be pointed out these trees are not independent of each other due to their datasets having shared the same *C-mos* gene sequences—the dataset for the former tree consisted solely of *C-mos* sequences (373 bp) while the dataset for the latter tree consisted of four nDNA genes including *C-mos* sequences (3,019 bp total). Although it is conceivable that the same placement of *D. mitella* in both trees was determined by the *C-mos* sequences, I find this scenario unlikely given the relative dataset sizes. This question of dataset independence aside, we can favor the nDNA placement of *D. mitella* on the basis of relative dataset sizes—the nDNA tree in Fig. 14E contains more characters (and loci) than the datasets for the mtDNA trees (Criterion 8; Table 2).

**Figure 15:**
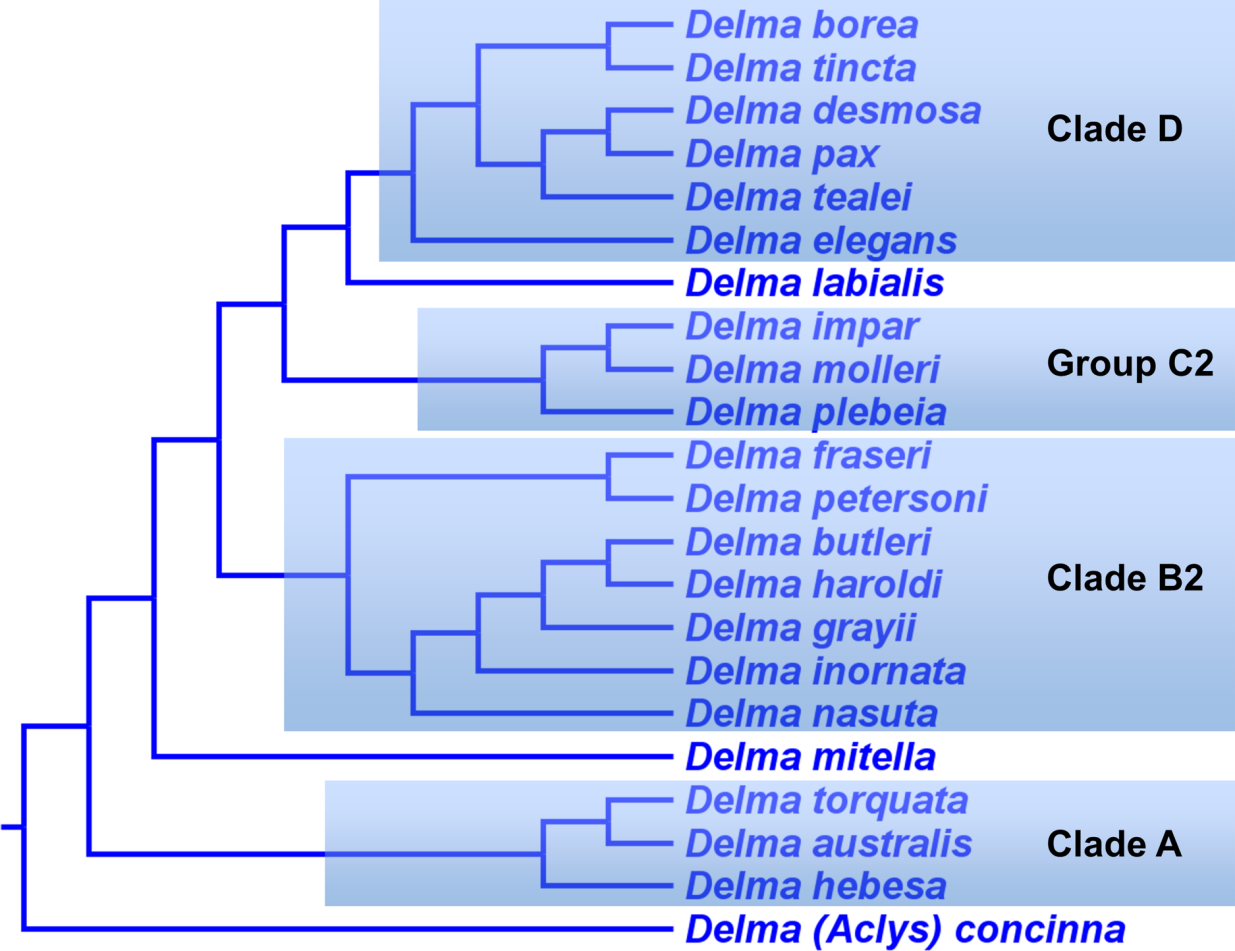
A new hypothesis for the phylogeny of *Delma*. Each species group is shaded in blue. See Table 3 for a list of species in each group. *Delma (Aclys) concinna*, *D. labialis*, and *D. mitella* are divergent single-species lineages that do not belong to any group.

Figure 15 shows a novel tree hypothesis for the genus *Delma*, which is based on the evidence considered above. Note that the position of *D*. (*Aclys*) *concinna* as the presumed sister lineage to all other species of *Delma* means that this species could be recognized as a species of *Delma* (following current practice) or, alternatively, it could be returned to its original monotypic genus of *Aclys* (Kluge, 1974). Although current evidence favors placement of *concinna* as the basal lineage in *Delma*, future phylogenomic studies of pygopodids may place *concinna* in other locations of the *Delma* phylogeny as some earlier phylogenetic hypotheses have done (Figs. 14B and 14C). Accordingly, I recommend continued recognition of *Delma concinna*.

### Genus *Lialis*

Two species represent this genus, *Lialis burtonis* and *L. jicari* GA Boulenger (Kluge, 1974). The former species is distributed across Australia and New Guinea, while the latter is restricted to New Guinea (Kluge, 1974). Owing to their near-identical appearance and unique and divergent morphology/ecology compared to other living pygopodids, it is not surprising that these two species have been placed as sister species in all four independent tree hypotheses (Figs. 5, 6, 7 and 11). Thus, there can be no doubt that *Lialis* is monophyletic (Criterion 6; Table 2).

### Genus *Pletholax*

Storr (1978) split the monotypic *Pletholax gracilis* into two geographically separated subspecies: *P. g. gracilis* from the central west coast of Western Australia and *P. g. edelensis* GM Storr from the Shark Bay region well north of the nominate form’s distribution (Storr, Smith & Johnstone, 1990). Kealley et al. (2020) re-evaluated the taxonomic status of *P. g. edelensis* in light of morphological and molecular evidence. These authors concluded that both subspecies of *Pletholax* represent distinct species and thus they elevated *P. g. edelensis* to full species status. Accordingly, *Pletholax* is now comprised of two species: *P. gracilis* and *P. edelensis* GM Storr.

### Genus *Pygopus*

The genus *Pygopus* currently contains five species, or six if the monotypic *Paradelma orientalis* is subsumed into this group according to the recommendation by *K76*. In Kluge (1974), only two species of *Pygopus*—*P. nigriceps* and *P. lepidopodus*—were recognized. But Kluge (1974) believed that other species of *Pygopus* were waiting to be discovered, as he also recognized two subspecies of *P. nigriceps* (i.e., *P. n. nigriceps* and *P. n. schraderi* GA Boulenger) owing to morphological variation between populations. Later, James, Donnellan & Hutchinson (2001) elevated *P. n. schraderi* to species status and described a new species, *P. steelescotti* BH James, SC Donnellan, MN Hutchinson, based on morphological, distributional, and molecular phylogenetic evidence. Their allozyme tree suggested that *P. schraderi* and *P. steelescotti* were sister species, while *P. nigriceps sensu stricto* was placed as their sister lineage (James, Donnellan & Hutchinson, 2001). However, because these workers had rooted the tree with *P. lepidopodus*, a congener that was assumed to be outside this species group, only a partial test of *P. nigriceps* group monophyly can be made. If we thus look at their tree as an unrooted tree, then we can see that a single branch separates the cluster containing *P. nigriceps sensu stricto*, *P. schraderi*, and *P. steelescotti* from the outgroup *P. lepidopodus*. Not surprisingly, their allozyme tree is evidence consistent with monophyly of the *P. nigriceps* species complex. Moreover, two other independent trees corroborate monophyly of the *P. nigriceps* group (Figs. 10 and 11; also see Fig. 3 in Oliver, Couper & Amey, 2010). Thus, monophyly of the *P. nigriceps* group is firmly established (Criterion 6; Table 2).

*Pygopus lepidopodus* also appears to be a complex of species rather than just a single widely distributed species. Based on molecular, morphological, and geographical evidence, Oliver, Couper & Amey (2010) described a distinctive population of *P. lepidopodus* from northeastern Queensland as a new species named *P. robertsi* PM Oliver, P Couper, A Amey. In addition to this new species description, Oliver, Couper & Amey (2010) also revealed the molecular and morphological diversity found across the geographic range of *P. lepidopodus*, variation that hints at the existence of other cryptic species in this complex. These authors used mtDNA to infer a phylogeny for various populations of *P. lepidopodus* and three recognized species of the *P. nigriceps* species complex. Moreover, because they had rooted their tree with exemplars of other pygopodid genera, they could test the monophyly of both species complexes. Their tree supported monophyly of both the *P. lepidopodus* and *P. nigriceps* species complexes (see Fig. 3 in Oliver, Couper & Amey, 2010).

Although the study by Oliver, Couper & Amey (2010) was aimed at analyzing *P. lepidopodus* diversity, their tree also yielded a hypothesis for the relationships among the members of the three-species *P. nigriceps* group. In contrast to the allozyme tree in James, Donnellan & Hutchinson (2001), the mtDNA tree in Oliver, Couper & Amey (2010) showed *P. nigriceps* and *P. steelescotti* as sister species with *P. schraderi* placed as their sister lineage (see Fig. 3 in Oliver, Couper & Amey, 2010). Given the apparent prevalence of mtDNA introgression that has been uncovered in pygopodids, it would not be surprising if this had also occurred between *P. nigriceps* and *P. steelescotti*, especially because their ranges contact other (see Fig. 3 in James, Donnellan & Hutchinson, 2001).

Further phylogenetic uncertainty about the relationships within the *P. nigriceps* complex is added when the concatenated mtDNA + nDNA tree from *B&O* is also considered. In this tree, *P. nigriceps* and *P. schraderi* were placed together as sister species while *P. steelescotti* was their sister lineage (Fig. 10). If we focus only on the *P. nigriceps* group, and examine which genes were sequenced for these three species, it appears as though the topology of their tree was largely determined by the nDNA signal in their data matrix. This conclusion is reached owing to the following observations: mtDNA gene (*ND2*) sequences for all three species were analyzed while nDNA sequences for only a single nDNA gene (*RAG1*) were analyzed for *P. nigriceps* and *P. schraderi*. If these *RAG1* sequences were omitted from the phylogenetic analysis, then presumably their tree would match the mtDNA-only results of Oliver, Couper & Amey (2010). This three-way tie among the competing topologies suggested by allozyme, mtDNA, and mtDNA + *RAG1* trees is broken when we look at the UCE trees in *SB&O* (Fig. 11). Both UCE topologies for the *P. nigriceps* group agree with the allozyme tree in James, Donnellan & Hutchinson (2001). Therefore, these two studies independently corroborate monophyly of *P. schraderi* and *P. steelescotti* (Criterion 6; Table 2).

The monophyly of the *Pygopus/Paradelma* group is beyond question given all the phylogenetic evidence supporting this grouping (Figs. 5, 6, 7, 9, 10 and 11; and see Fig. 3 in Oliver, Couper & Amey, 2010; Criterion 6; Table 2). However, there has been some question as to whether *Paradelma* warrants recognition as a monotypic genus or subgenus within *Pygopus*. The morphology-based tree in Kluge (1976; Fig. 2*)* showed *Paradelma* as the sister species to *P. nigriceps*, which led Kluge to subsume *Paradelma* into *Pygopus*. Since then, however, molecular studies have strongly corroborated the placement of *Paradelma* as the sister lineage to *Pygopus* (Figs. 6, 10 and 11; Criterion 7; Table 2), which suggests that the position of *Paradelma* in the morphology tree was incorrect. Accordingly, the justification for demoting *Paradelma* to subgeneric status has vanished and thus there is no reason why *Paradelma* cannot be recognized as a genus once again, especially given its unique morphological characteristics (see *K76*, p. 68). Given the strength of the evidence, *Paradelma* should be recognized as a pygopodid genus once again.

## Intergeneric relationships in the pygopodidae

Given the robust support for the monophyly of all five multispecies genera in the Pygopodidae—*Aprasia*, *Delma*, *Lialis*, *Pletholax*, and *Pygopus*, we can now turn our attention to the relationships among these major groups and the two monotypic genera, *Ophidiocephalus* and *Paradelma*. Unfortunately, there has been little agreement among tree hypotheses concerning the relationships among pygopodid genera *(JP&D; BB&J; B&O; SB&O; Kealley et al., 2020)*. This problem might stem from short internal branches that have been observed in family-level trees (see Fig. 11 in *JP*&*D* and Fig. 2 in *BB*&*J*). The shortness of these branches may reflect an early and rapid diversification episode early in the group’s history (*JP*&*D*).

To simplify the task of finding the best-supported intergeneric tree hypothesis for pygopodids, we will take as a given that *Paradelma* is the sister lineage to *Pygopus* owing to strong support for this relationship. Moreover, because of the strong evidence suggesting *Delma* is the sister group to a clade containing all other pygopodid genera, the problem of inferring the pygopodid intergeneric relationships is reduced to finding the most probable five-taxon tree that is the sister group to *Delma*. However, inferring the correct rooted tree topology even for five lineages is a daunting challenge given that there are 105 possible rooted bifurcating trees for this number of taxa (Felsenstein, 2004).

**Figure 16:**
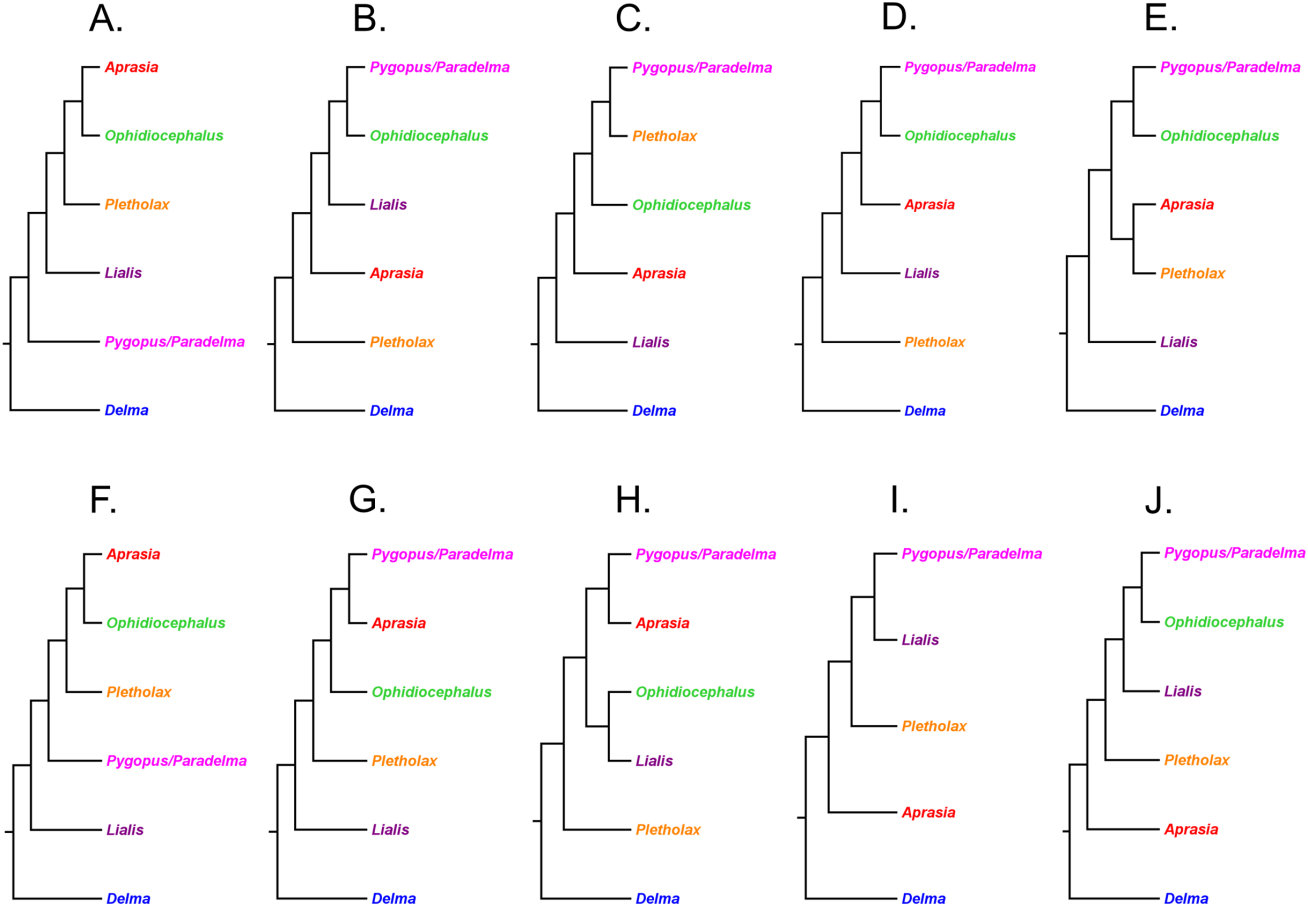
Hypotheses of phylogenetic relationships among pygopodid genera. (A) Cladogram derived from Fig. 5 (i.e., maximum parsimony tree based on 86 morphological characters). Modified version of Fig. 9 in Kluge (1976). Note, this same topology can also be derived from a Bayesian inference tree based on concatenated two mtDNA (*16S* and *ND2*) genes and one nDNA (*C-mos*) gene, and 86 morphological characters (see Fig. 6B in Jennings, Pianka & Donnellan, 2003) if outgroup species are removed and the tree is re-rooted along the branch leading to *Delma*. (B) Cladogram derived from Fig. 6 (i.e., maximum parsimony/maximum likelihood/Bayesian inference cladograms based on two mtDNA [*16S* and *ND2*] genes). After Fig. 2 in Jennings, Pianka & Donnellan (2003) if outgroup species are removed and the tree is re-rooted along the branch leading to *Delma*. (C) Cladogram derived from Fig. 7 (i.e., maximum parsimony/maximum likelihood/Bayesian inference cladograms based on the nuclear *C-mos* gene). After Fig. 4 in Jennings, Pianka & Donnellan (2003) if outgroup species are removed and the tree is re-rooted along the branch leading to *Delma*. (D) Cladogram derived from maximum parsimony tree based on concatenated two mtDNA (*16S* and *ND2*) genes and one nDNA (*C-mos*) gene. After Fig. 5A in Jennings, Pianka & Donnellan (2003) if outgroup species are removed and the tree is re-rooted along the branch leading to *Delma*. (E) Cladogram derived from maximum likelihood/Bayesian inference trees based on concatenated two mtDNA (*16S* and *ND2*) genes and one nDNA (*C-mos* gene) gene. After Figs. 5B and 5C in Jennings, Pianka & Donnellan (2003) if outgroup species are removed and each tree is re-rooted along the branch leading to *Delma*. (F) Cladogram derived from maximum parsimony tree based on concatenated two mtDNA (*16S* and *ND2*) genes and one nDNA (*C-mos*) gene, and 86 morphological characters. After Fig. 6A in Jennings, Pianka & Donnellan (2003) if outgroup species are removed and the tree is re-rooted along the branch leading to *Delma*. (G) Cladogram derived from Fig. 9 (i.e., maximum likelihood/Bayesian inference trees based on four nDNA [*C-mos, DYNLL1, RAG1*, and *MXRA5*] genes). Modified version of Fig. 2 in Brennan, Bauer & Jackman (2016). (H) Cladogram derived from Fig. 10 (i.e., Bayesian inference tree based on concatenated one mtDNA [*ND2*] and six nDNA [*C-mos, DYNLL1, PDC, RAG1*, RAG2, and *ACM4*] genes). Modified version of Fig. 1 in Brennan & Oliver (2017). (I) Cladogram derived from Fig. 11 (i.e., multispecies coalescent [MSC] species tree/concatenated data maximum likelihood cladogram inferred from 4,268 UCE loci). Modified version of Fig. 4 in Skipwith, Bi & Oliver (2019). (J) Cladogram derived from maximum likelihood tree based on concatenated one mtDNA (*ND2*) and two nDNA (*PRLR* and *PTPN12*) genes. Modified version of Fig. 7 in Kealley et al. (2020).

If we reexamine the published phylogenies of pygopodid lizards, we find a total of nine different trees that contained at least one member of each genus plus one tree that contained all genera except *Ophidiocephalus* (Fig. 16). A significant problem with this sample of trees is that the majority of them do not represent independent tree inferences owing to shared data among them. For example, trees in Figs. 16B, 16D, 16E, 16F, 16H, and 16J were inferred from datasets containing the same (or largely the same) set of mtDNA sequences. Also, the trees in Figs. 16C, 16D, 16E, 16F, 16G, and 16H shared the same *C-mos* nuclear gene sequences. To simplify this analysis and ensure more robust inter-tree comparisons, I focused on the following three independent trees: morphology tree in *K76* (Fig. 16A), concatenated nDNA (i.e., *C-mos, DYNLL1*, *RAG1*, and *MXRA5* genes) tree in *BB*&*J* (Fig. 16G), and the concatenated mtDNA + nDNA (i.e., *PRLR* and *PTPN12* genes) tree in Kealley et al. (2020; Fig. 16J). Although the mtDNA-only tree (Fig. 16B) represents an independent tree inference, the substantial data overlap with the mtDNA + nDNA tree (Fig. 16J) required me to choose one tree and discard the other from this analysis. I therefore selected the latter tree because it had been inferred from a larger number of DNA sites than the former tree (1,706 vs. 2,415 bp). Likewise, because the nDNA tree (Fig. 16C) and concatenated nDNA tree (Fig. 16G) both employed the nuclear *C-mos* gene, I chose the latter tree because it was based on a much larger dataset (373 vs. 3,019 bp) compared to the former tree. Two key qualities of these three selected trees include: each represents an independent tree inference and each contains all generic lineages.

Unfortunately, these three trees (Figs. 16A, 16G and 16J) show much disagreement with each other. Nonetheless, we can search for common lineage placements among them, which might lead us to the true tree. First, we can see that all three trees have a ladderized structure with a two-lineage clade on “top” (in these figures). We will assume that the true tree has this same shape since there are no other tree shapes suggested by the sample. Second, notice that the top clade of each tree contains three different sister lineage pairings: *Aprasia* and *Ophidiocephalus* (Fig. 16A), *Aprasia* and *Pygopus*/*Paradelma* (Fig. 16G), and *Ophidiocephalus* and *Pygopus*/*Paradelma* (Fig. 16J). If we assume a bifurcating tree, then only two of these taxa can be sister lineages to each other, though it is not obvious which two they could be. The alternative placements for these three generic lineages suggest that *Ophidiocephalus* may instead be the lineage that is sister to an *Aprasia* and *Pygopus*/*Paradelma* sister pair (Fig. 16G), or that *Aprasia* or *Pygopus*/*Paradelma* might be placed deeper in the tree (Figs. 16A and 16J). Last, because *Pletholax* occupies the same position in two of the three trees (Figs. 16G and 16J), we will accept this placement.

If we could determine which generic lineage—*Aprasia*, *Pygopus/Paradelma*, or *Ophidiocephalus*—is incorrectly placed, then it may be possible to elucidate the remainder of the intergeneric tree. The topology based on the two UCE trees (Fig. 16I) may help in this regard because it too represents an independent tree inference. Although the missing *Ophidiocephalus* lineage in the UCE tree precludes lineage-by-lineage comparisons between this and the trees in Fig. 16, the UCE tree hypothesis may still provide helpful clues on the most probable locations for unplaced taxa. One obvious clue is that *Aprasia* is located in a relatively basal position in this tree while *Pygopus*/*Paradelma* is located in a relatively derived place or “top” part of the ladderized tree (Fig. 16I)—a similar topological pattern seen in the mtDNA + nDNA tree (Fig. 16J). Thus, if we consider *Aprasia* to be the sister lineage to the other four lineages, then *Pygopus*/*Paradelma* would appear to be the sister group to *Ophidiocephalus*, with *Lialis* being their sister lineage (Fig. 16J). Remarkably, if we were to insert *Ophidiocephalus* as the sister lineage to *Pygopus/Paradelma* in the UCE tree (Fig. 16I), then the resulting topology would match the mtDNA + nDNA tree in Fig. 16J.

Although support for this hypothetical tree is contingent on *Ophidiocephalus* being the sister lineage to *Pygopus*/*Paradelma*, there is evidence to support this idea. The *Ophidiocephalus* and *Pygopus/Paradelma* sister lineage relationship not only appeared in the mtDNA-only (Fig. 16B) and mtDNA + nDNA (Fig. 16J) trees, but this clade was also was recovered in the mtDNA + nDNA trees in *JP*&*D* (Figs. 16D and 16E). Indeed, results of hypothesis testing in *JP*&*D* supported the sister relationship between the *Ophidiocephalus* and *Pygopus*/*Paradelma* clade. Thus, by assuming that *Ophidiocephalus* is the sister lineage to the *Pygopus*/*Paradelma* clade in the UCE tree (Fig. 16I), we can find complete agreement with another independent tree hypothesis (Fig. 16J). Therefore, I suggest that the tree topology shown in Fig. 16J represents the best current hypothesis of pygopodid intergeneric relationships. However, it must be emphasized that even though some aspects of this intergeneric tree hypothesis are robust (i.e., the root location, basal position of *Delma*, sister lineage relationship between *Pygopus* and *Paradelma*), the relationships among the other generic lineages are weakly supported.

## A composite phylogeny for the pygopodidae

**Figure 17:**
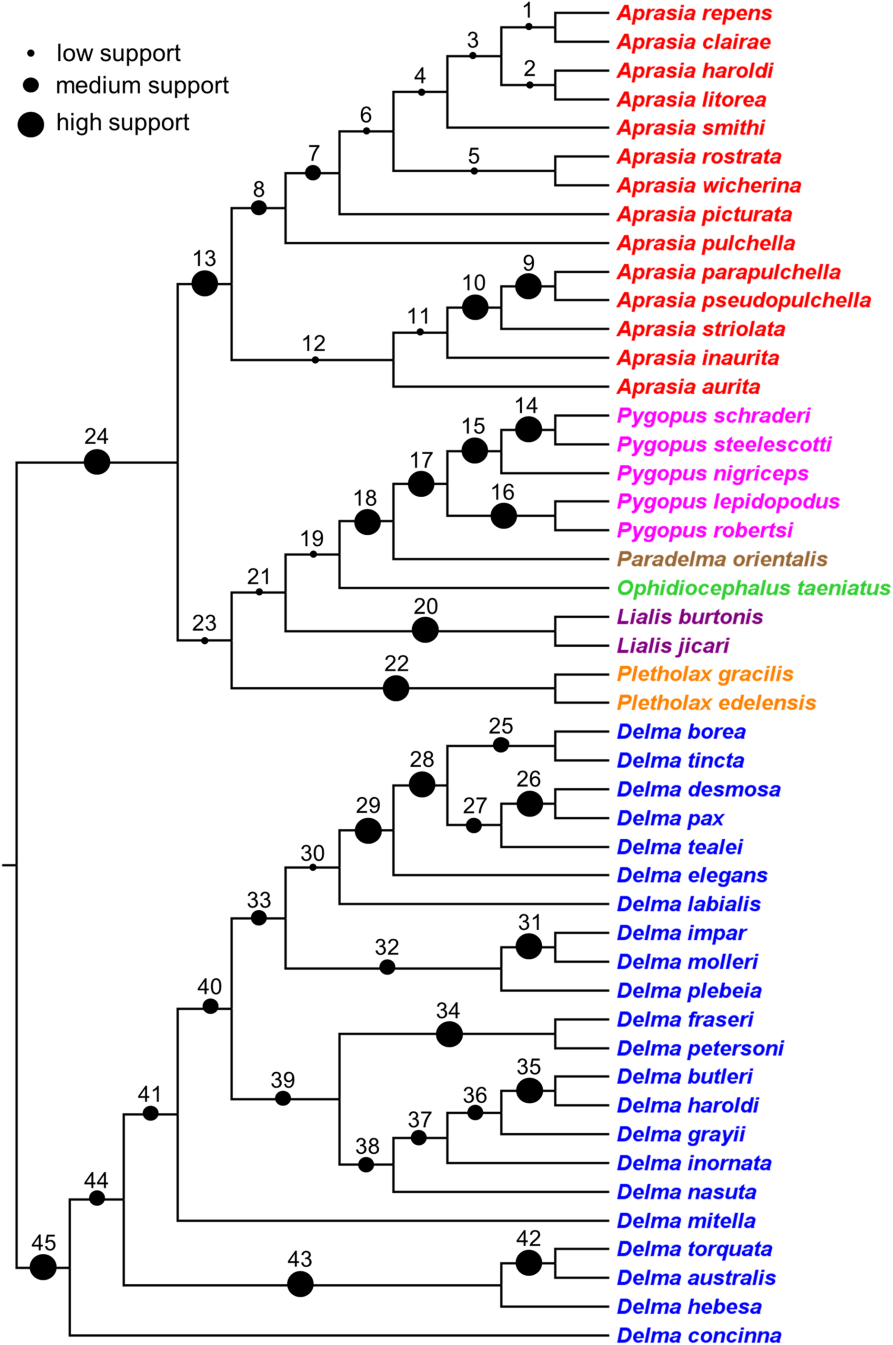
A composite tree for 47 species of pygopodid lizards. Each branch that defines a clade is numbered and the solid dark circles indicate one of three levels of clade support: small-sized circle (“low support”) indicates that the clade was supported by a single independent tree; medium-sized circle (“medium support”) indicates that the clade was supported by a multispecies coalescent (MSC) species tree based on genome-wide data or was corroborated by two independent trees but missing taxa or strongly conflicting evidence creates some uncertainty about this clade; and large-sized circles (“high support”) indicates that the clade was supported by multiple independent trees or the clade is comprised by two species that were formerly described as a single species. Table S1 provides descriptions of each clade and the bases for clade-support designations. Colors correspond to different genera.

At the time *K76* published the first phylogenetic hypothesis for the Pygopodidae there were 30 recognized species in this lizard family. Since then an additional 17 species have been discovered, which brings the total known species richness of this group to 47 species. Having reviewed primary literature studies concerned with pygopodid phylogeny, we can synthesize a composite tree that includes all valid species in this family. Notice that nearly half (44%) of the 45 total clades within this tree have relatively high empirical support, whereas the remaining clades have less support (Fig. 17; Table S1). Thus, major portions of the pygopodid family tree have now been corroborated by multiple independent tree hypotheses. However, it should be emphasized that these measures of empirical clade support are not statistically based and thus they should be regarded as being more subjective measures of clade support.

Monophyly of *Aprasia*, *Delma*, *Lialis*, and *Pygopus* is well established (Fig. 17; Table S1). Moreover, placement of the tree’s root has also been corroborated by multiple independent trees thereby further reinforcing the viewpoint that the *Delma* group is not only monophyletic, but is the sister clade to a superclade containing the other six pygopodid genera (Fig. 17). Dissecting these results further, we see that support for these monophyletic groups came from varied types of data including morphology, mtDNA, and several independent nDNA datasets. Although mtDNA sequence datasets contain orders of magnitude fewer characters than today’s phylogenomic datasets, it is astonishing that mtDNA trees were able to independently recover these monophyletic generic groups as well as point out the likely root location. Thus, mtDNA trees were as informative, in this regard, as any other independent tree including those based on thousands of UCE loci. However, trees inferred from mtDNA and from all other datasets in studies of pygopodid phylogeny failed to elucidate all of the intergeneric relationships. Indeed, branches 19, 21, and 23 in Fig. 17 remain poorly supported despite many attempts to reconstruct them (Fig. 16).

Empirical support for relationships within genera varied considerably. For instance, the majority of branches inside *Aprasia* have only single-tree support (allozyme or mtDNA), though morphology and mtDNA trees agree on branches 9 and 10 (Fig. 17; Table S1). In contrast, all branches within *Pygopus* and *Lialis* are corroborated by multiple independent trees: allozyme, mtDNA, and two independent nDNA trees corroborated all branches within *Pygopus* while morphological, mtDNA, and nDNA trees corroborated *Lialis* monophyly (Fig. 17; Table S1). Although the two-species *Pletholax* clade was only supported by a tree based on a concatenated mtDNA + nDNA dataset, there is no doubt (like the two species of *Lialis*) that they are sister species given their identical appearance and extremely divergent morphology compared to other extant pygopodids. The *Delma* clade shows a mix of branch support designations. For example, clades defined by branches 26, 28, 29, 31, 34, 35, 42, and 43 were all corroborated by mtDNA and/or nDNA trees, while branches 26 and 31 were also independently supported by allozyme and morphology trees, respectively (Fig. 17; Table S1).

Unfortunately, instability in the placement of *D. labialis* across independent trees limited many otherwise corroborated branches (i.e., 30, 33, 40, 41, and 44) to receiving low to medium instead of high support designations (Fig. 17; Table S1). Another species, *D. inornata*, also created phylogenetic uncertainty within *Delma* but not because of placement instability; instead it was because this species was missing in some trees. According to the statistically strong nDNA results in *BB*&*J*, *D. inornata* belongs to a five-species clade (i.e., defined by branch 38 in Fig. 17). In the subsequent phylogenomic study by *SB&O*, their MSC-UCE tree contained a similar monophyletic group except *D. inornata* was absent from the tree. Accordingly, branch 38 could only be partially corroborated by the UCE tree, resulting in a designation of medium rather than high branch support.

## Conclusions

The lizard family Pygopodidae contains 47 recognized species in seven genera. Multiple independent phylogenetic trees corroborate several key features of the pygopodid family tree including: monophyly of *Aprasia*, *Delma*, *Lialis*, and *Pygopus*; a sister group relationship between *Delma* and all other genera; monophyly of the *Aprasia repens* and *Delma tincta* groups; and the sister-genus relationship between *Paradelma* and *Pygopus*. Two taxonomic conclusions are reached here: the established sister relationship between *Pygopus* and *Paradelma* provides the basis for re-recognition of *Paradelma* as a pygopodid genus rather than as a subgenus under *Pygopus*; and the recommendation by Kluge (1976) to consider the monotypic *Aclys concinna* as a member of the *Delma* clade should be followed until future studies can clarify the position of *D. concinna*—most likely as either the sister lineage to *Delma* or a species nested within *Delma*. The placements of several clades and single species lineages in the pygopodid family tree are either controversial or have low empirical support due to several factors: (1) ancient hybridization, including at least five cases of mito-nuclear discordance, may have confounded attempts to recover some relationships in the pygopodid species tree using molecular data; (2) the placements of several “long-branch” species including *D. concinna*, *D. labialis*, and *D. mitella* in the *Delma* clade are still not established; and (3) the majority of intergeneric relationships remain poorly supported, evidently due to short internal branches that may reflect the rapid diversification of generic lineages early in the group’s history. The composite phylogeny for the Pygopodidae produced here can serve as a hypothesis in future phylogenomic studies.

## Supplemental Information

10.7717/peerj.11502/supp-1 Supplemental Information 1 Summary of evidence for each chosen pygopodid clade. Clade support definitions: “low support” indicates that the clade was supported by a single independent tree; “medium support” means that the clade was found in a multispecies coalescent (MSC) species tree based on large numbers of genome-wide loci, or was corroborated by two independent trees but missing taxa or conflicting evidence raises uncertainty; and “high support” denotes the clade was supported by multiple independent trees, or the clade is comprised of two species that were formerly described as a single species. See also Fig. 17.

## Additional Information and Declarations

### Data Availability

The following information was supplied regarding data availability: The summary of evidence for each chosen pygopodid clade is available in the Supplemental File.
